# AutoMorphoTrack: A modular framework for quantitative analysis of organelle morphology, motility, and interactions at single-cell resolution

**DOI:** 10.1101/2025.07.19.665650

**Published:** 2026-02-02

**Authors:** Armin Bayati, Jackson G. Schumacher, Xiqun Chen

**Affiliations:** 1 Department of Neurology, Massachusetts General Hospital and Harvard Medical School, Boston, MA 02129, USA; 2 Aligning Science Across Parkinson’s (ASAP) Collaborative Research Network, Chevy Chase, MD 20815, USA

## Abstract

Quantitative imaging of organelle dynamics provides crucial insights into cellular function, state, and organization; however, existing analysis workflows often require advanced coding expertise and multiple software tools. AutoMorphoTrack is an open-source Python toolkit that automates organelle detection, morphology classification, motility tracking, and colocalization from multichannel fluorescence microscopy image stacks. The platform includes adaptive segmentation, organelle trajectory reconstruction, and pixel-level overlap quantification within a unified, reproducible framework that can be executed as an interactive Jupyter notebook, a modular Python package, or through AI-assisted natural-language commands. Each analysis step outputs publication-ready images, time-lapse videos, and standardized quantitative data tables. To complement the main pipeline, an accompanying script—AMTComparison.py—is provided to demonstrate how AutoMorphoTrack’s outputs can be extended for comparative analysis across individual neurons or experimental conditions. Together, these tools provide an accessible and framework for high-content, reproducible quantification of subcellular morphology, motility, and interactions at single-cell resolution.

## Graphical Abstract

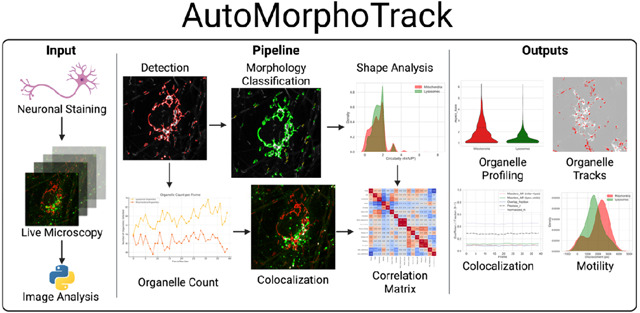

## Introduction

Quantitative live-cell imaging has become indispensable for studying subcellular organization, revealing how organelles interact, adapt, and reorganize in response to physiological and pathological cues ^1^. In neurons, organelle dynamics reflect the balance between energy production, trafficking, and degradation, processes that are tightly regulated through mitochondrial morphology, lysosomal positioning, and their physical and functional interactions ^2,3^. While microscopy captures these processes at high temporal and spatial resolution, translating those images into interpretable, quantitative information remains challenging. Most analysis workflows, although very powerful in their outputs, require coding/programming knowledge or rely on proprietary software that lacks transparency and reproducibility ^4–6^.

AutoMorphoTrack was developed to bridge this divide by providing an open-source, automated, and reproducible image analysis framework specifically designed for cell biologists. The toolkit performs every major stage of organelle quantification: detection, classification, tracking, motility measurement, and colocalization, all within a flexible structure that can be executed in three ways: as a step-by-step Jupyter notebook for interactive analysis, as a standalone Python package for batch processing, or through AI-assisted natural-language prompts. Each function produces both visual and quantitative outputs, allowing users to verify segmentation accuracy, track organelle behavior across time, and extract shape and motion parameters.

The central goal of AutoMorphoTrack is transparency without complexity. Every processing step is exposed to the user through editable parameters—such as threshold sensitivity, eccentricity, and minimum object size—so that segmentation and classification can be optimized without altering the source code. The system outputs standardized image and data formats, including .png, .mp4, and .csv, ensuring compatibility with downstream visualization or statistical tools. This standardization allows results from different experiments or cell types to be compared with ease.

To illustrate how AutoMorphoTrack data can be extended beyond single-sample analysis, we provide an independent companion script, AMTComparison.py, which demonstrates a flexible approach to comparing results between neurons or experimental conditions. Rather than embedding statistical testing directly into the package, this separate script shows how exported outputs can be analyzed using customizable statistical methods, including t-tests, Welch’s tests, or nonparametric alternatives. This design preserves analytical freedom while maintaining full compatibility with external platforms such as GraphPad Prism, R, or custom Python workflows.

By combining automation, reproducibility, and flexibility, AutoMorphoTrack transforms high-resolution microscopy into a quantitative, interpretable, and extensible dataset. It provides intricate information for examining how organelle morphology, movement, and interaction differ across samples/conditions, allowing computational analysis to provide biological insight.

## Methods

### Software Architecture and Workflow Overview

AutoMorphoTrack was developed in Python (≥ 3.9) as an image-analysis framework that performs organelle detection, morphological classification, motility tracking, and colocalization within a single, reproducible pipeline (Table 1). Each analytical component is organized as an independent module (Table 2) that can be executed sequentially to generate standardized outputs (Table 3) or imported individually for custom workflows. All modules rely on open-source libraries—NumPy (RRID:SCR_008633), pandas Pandas (RRID:SCR_018214), matplotlib Matplotlib (RRID:SCR_008624), seaborn seaborn (RRID:SCR_018132), OpenCV (RRID:SCR_015526), scikit-image (RRID:SCR_021142), SciPy (RRID:SCR_008058), and tifffile (RRID:SCR_023338)—and run efficiently on most CPU-based computers.

The pipeline can be executed as (i) an interactive Jupyter Notebook (RRID:SCR_018315), (ii) a Python package for automated batch analyses, or (iii) an AI-assisted natural-language interface that interprets plain-text prompts. Each mode implements an identical coding language to ensure that results are comparable across execution environments.

### Input Data and Preprocessing

AutoMorphoTrack is designed to analyze multichannel fluorescence microscopy data containing two distinct organelle populations. Although originally optimized for mitochondria (Channel 0) and lysosomes (Channel 1), the framework is adaptable to any pair of fluorescently labeled structures, such as endosomes, peroxisomes, autophagosomes, Golgi vesicles, or other dynamic compartments, by redefining the input channels and adjusting detection parameters.

Input files should be supplied as multichannel .tif image stacks obtained from live-cell microscopy. Each stack is parsed to verify dimensional consistency and channel count prior to analysis. Individual channels are converted to 8-bit grayscale to standardize intensity across datasets, and optional spatial upscaling (typically 2×) can be applied to improve segmentation accuracy in low-resolution or dimly labeled samples.

### Adaptability

User-defined parameters enable flexible adaptation to diverse imaging sources. The minimum particle size parameter excludes background noise and camera artifacts; the threshold sensitivity factor (thr_factor) fine-tunes segmentation stringency relative to the Otsu threshold; and channel assignment variables (MITO_CHANNEL, LYSO_CHANNEL) allow users to specify which channels correspond to the organelle of interest. This parameter transparency ensures that AutoMorphoTrack can be applied across imaging systems and experimental contexts—from conventional confocal microscopy to high-throughput spinning-disk or super-resolution platforms—without modifying the underlying source code, making it easier for biologists to utilize it in their everyday experiments.

Preprocessed frames serve as the standardized input for all subsequent analytical steps, generating consistent data structures that enable direct comparison across organelle types, cell models, or experimental conditions.

### Cell Culturing and Preparation

Human induced pluripotent stem cell (iPSC)–derived Neural Progenitor Cells (NPCs) were obtained from STEMCELL Technologies (Catalog #200–0620) and expanded in Neural Progenitor Medium (STEMCELL, Catalog #05833) according to the manufacturer’s instructions. After two passages, NPCs underwent midbrain differentiation using the Midbrain Neuron Differentiation Kit (STEMCELL, Catalog #100–0038) supplemented with 200 ng/mL Human Recombinant Sonic Hedgehog (Shh; STEMCELL, Catalog #78065) for 7 days. Dopaminergic NPCs were then plated on poly-D-lysine and Matrigel–coated glass-bottom 6-, 24-, or 96-well plates (Thermo Scientific, Nunc) for Western blotting, fluorescence microscopy, and quantitative imaging, respectively.

Differentiated NPCs were matured into neurons using the Midbrain Neuron Maturation Kit (STEMCELL, Catalog #100–0041) for two weeks. Following maturation, neurons were maintained in BrainPhys Neuronal Medium supplemented with the SM1 Kit (STEMCELL, Catalog #05792). For each biological replicate, neurons derived from independent differentiation events were plated on glass coverslips (Thomas Scientific, Catalog #1217N79) pre-coated with Matrigel (Corning, Catalog #356234) in 12- or 24-well plates. After completing each replicate, new NPC stocks were thawed and differentiated independently to ensure biological reproducibility.

Fluorescence imaging was performed using a Nikon C2 Confocal Microscope equipped with NIS-Elements software (Nikon, RRID: SCR_014329). For live-cell imaging, dopaminergic neurons were plated on glass-bottom dishes (MatTek, P35G-1.5–14-C) and matured for two weeks. Prior to imaging, neurons were stained with MitoTracker Red (Invitrogen, M7510) and LysoTracker Green (Invitrogen, L7526) for 30 minutes in BrainPhys medium. Live imaging was conducted using a Nikon C2 confocal microscope equipped with a stage-top environmental chamber (37 °C, 5% CO ) and a 63× oil-immersion objective. Time-lapse images were acquired at 5 s intervals for 40 consecutive frames in multichannel mode and exported as .tif stacks for downstream processing with AutoMorphoTrack.

### Step 1 – Organelle Detection

Segmentation is performed using adaptive Otsu thresholding combined with morphological opening, small-object removal, and border clearing. Detected objects are stored as binary masks for mitochondria and lysosomes. Corresponding color overlays (Mito_Frame0.png, Lyso_Frame0.png) and detection videos (*_Detection.mp4) are produced automatically (Table 2). These visual outputs allow real-time verification of segmentation accuracy and are reused for subsequent morphology, tracking, and colocalization analyses.

### Step 2 – Lysosome Counting

The lysosomal counting module enumerates and labels individual lysosomes per frame, providing a quantitative measure of vesicle abundance over time. Outputs include labeled images (Lyso_Frame0_Count.png), temporal plots (Lyso_Count_Plot.png), and the tabulated dataset Lysosome_Counts.csv. Together, these files lysosomal turnover and temporal heterogeneity, forming the first dynamic parameter set in the pipeline (Table 2, Step 2).

### Step 3 – Mitochondrial Morphology Classification

Mitochondrial networks are quantified by classifying individual mitochondria as elongated or punctate based on user-adjustable thresholds for area and eccentricity. The resulting labeled frame (Morphology_Frame0_Labeled.png) and summary tables (Morphology_Summary.csv) provide a rapid overview of morphological states across time. Because all parameters are adjustable, users can tune detection sensitivity to match imaging contrast or magnification, thereby ensuring consistency across experiments.

### Step 4 – Shape Feature Extraction

Beyond binary classification of mitochondria, AutoMorphoTrack computes shape descriptors—area, eccentricity, circularity, solidity, aspect ratio, and orientation—for every segmented organelle. The outputs (Mito_ShapeMetrics.csv, Lyso_ShapeMetrics.csv) provide frame-by-frame quantification of structural properties. AutoMorphoTrack then generates plots (Shape_ViolinPlots.png) that summarize morphological properties within and across organelle populations.

### Step 5 – Organelle Tracking and Motility Analysis

To capture organelle dynamics, coordinates from consecutive frames are linked using nearest-neighbor matching implemented via scipy.spatial.cKDTree. This approach reconstructs trajectories for each detected organelle, from which total displacement and mean velocity are computed. This step exports individual coordinate tables (*_Tracks.csv), scatter and distribution plots (Motility_Scatter.png, Motility_Distributions.png), and cumulative overlays (Cumulative_Mito.png, Cumulative_Lyso.png). These metrics describe the spatial range and kinetics of organelle movement.

### Step 6 – Colocalization Analysis

Colocalization between mitochondria and lysosomes is quantified using both Manders’ coefficients (M1, M2) and the Pearson correlation coefficient, computed directly from binary masks to ensure spatial precision. Visual overlays highlight overlapping pixels in bright blue, while numerical results are stored in Colocalization.csv and summarized graphically in Colocalization_MetricsPlot.png. The consistent data structure across analyses facilitates direct integration with motility and morphology outputs.

### Step 7 – Integrated Correlation Summary

All per-frame and per-object datasets are merged into an integrated summary table (Integrated_Merged_Data.csv) using pandas.merge(). Pearson correlation coefficients are calculated among morphology, motility, and colocalization parameters to generate a comprehensive overview of organelle behavior. The resulting heatmap (Integrated_CorrelationMatrix.png) reveals relationships such as whether elongated mitochondria exhibit unique motility or colocalization profiles compared to punctate ones.

### Step 8 – Comparative Analysis (Optional External Script)

While AutoMorphoTrack explicitly focuses on generating quantitative descriptors and outputs, comparative analyses between cells or experimental conditions can be conducted using the companion script AMTComparison.py. This external code illustrates how users can extend AutoMorphoTrack data into statistical frameworks of their choice. The script automatically loads exported .csv files generated by AutoMorphoTrack, produces violin plots for key metrics, and selects statistical tests (Student’s t, Welch’s, or Mann-Whitney U) based on normality and variance. It reports effect sizes (Cohen’s d, Hedges’ g, or rank-biserial r) alongside significance. Because optimal comparative strategies depend on study design, this step remains optional and modular.

### Software Organization and Package Structure

The internal architecture of AutoMorphoTrack consists of discrete Python modules that correspond to each analytical stage (Table 2). The core modules (detection.py, morphology.py, tracking.py, motility.py, colocalization.py, and summary.py) define the main computational pipeline, while supportive scripts (utils.py, lyso_count.py, shape_features.py) manage input/output parameters, visualization, and shared functions. The optional AMTComparison.py script is distributed with the manuscript as an independent example of downstream analysis. Data analysis following the use of AutoMorphoTrack is entirely dependent on the user’s preference; therefore, the AMTComparison.py is not integrated into the AutoMorphoTrack package.

### Summary of Outputs, Functions, and Package Design

Comprehensive overviews of AutoMorphoTrack’s outputs, functional steps, and module organization are provided in Table 1, Table 2, and Table 3. Together, these tables detail how the code converts raw multichannel image stacks into structured, quantitative datasets ready for visualization, comparison, and interpretation.

### AI-Assisted Execution and Natural-Language Interface

AutoMorphoTrack and its accompanying comparison script (AMTComparison.py) were designed for seamless integration with large-language-model (LLM)–based chat interfaces, enabling users to execute and modify analyses through natural-language commands rather dealing with the coding languages directly. Both the Jupyter notebook and Python package versions can be used interactively with AI chatbots (such as ChatGPT, GPT-4, or similar LLM platforms) that support Python code interpretation and file manipulation.

Through these interfaces, users can request specific analytical actions, for example, “segment mitochondria and lysosomes from this TIFF stack,” “calculate velocity distributions,” or “compare morphology metrics between neurons.” The chatbot interprets the prompt, executes the corresponding code block from the AutoMorphoTrack notebook or package, and returns the generated visual or quantitative outputs. This interaction provides an intuitive layer of automation that allows experimental biologists with limited coding experience to perform reproducible, high-content image analyses without modifying source code manually.

This AI-assisted execution mode maintains full reproducibility because every operation is logged within the notebook environment, and the underlying Python commands remain visible to the user. By combining open-source design with conversational accessibility, AutoMorphoTrack provides an interactive, educational, and reproducible framework for computational image analysis.

## Results

AutoMorphoTrack produces a sequence of results that quantify the morphology, dynamics, and interactions of subcellular organelles at single-cell resolution. Each analytical stage generates both visual and quantitative outputs that can be interpreted independently or as part of an integrated cellular profile. The following results illustrate the complete workflow applied to individual image stacks and its extension to comparative analyses between two image stacks. The application of AutoMorphoTrack to additional image stacks, along with the corresponding outputs, can be found in Figures S1-8.

### Detection, Counting, and Morphological Classification

**Figure 1.**
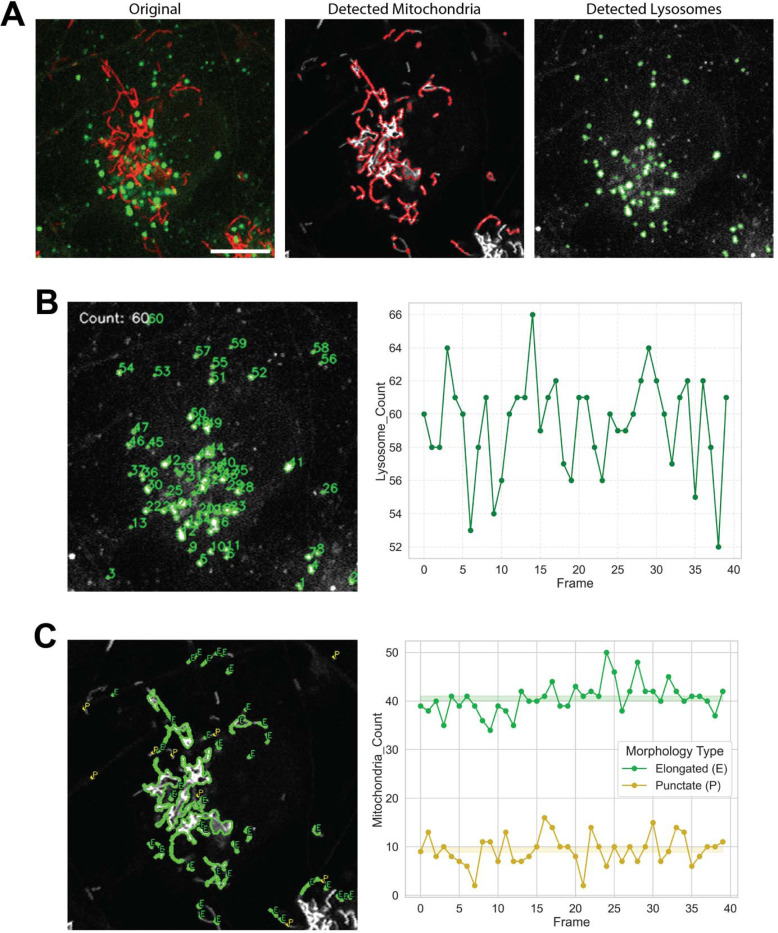
Automated detection, lysosomal counting, and mitochondrial morphology classification. (**A**) Composite multichannel fluorescence image showing mitochondria and lysosomes followed by independent detection maps for mitochondria. Scale bar = 10 μm. (**B**) Each lysosome is labeled and counted per frame, with a corresponding temporal plot illustrating vesicle abundance across the recording. (**C**) Mitochondrial morphology is classified as elongated or punctate based on area and eccentricity thresholds, shown as a labeled frame and quantitative summary chart. Together, these panels depict the initial detection and morphological analysis workflow implemented in AutoMorphoTrack.

The workflow begins with adaptive Otsu thresholding to segment mitochondria and lysosomes from multichannel fluorescence image stacks. A composite fluorescence image illustrates the original dataset, followed by independent detection maps showing mitochondria in red and lysosomes in green (Figure 1A). Each lysosome is labeled and enumerated per frame, producing a count map and a temporal plot of lysosome numbers across the recording (Figure 1B).

Mitochondrial morphology is automatically classified as elongated or punctate according to area and eccentricity thresholds. The resulting morphology map and quantitative summary chart capture both elongated and fragmented mitochondrial populations (Figure 1C). These steps collectively form the foundation of AutoMorphoTrack’s detection and morphology modules.

### Shape Profiling and Feature Extraction

**Figure 2.**
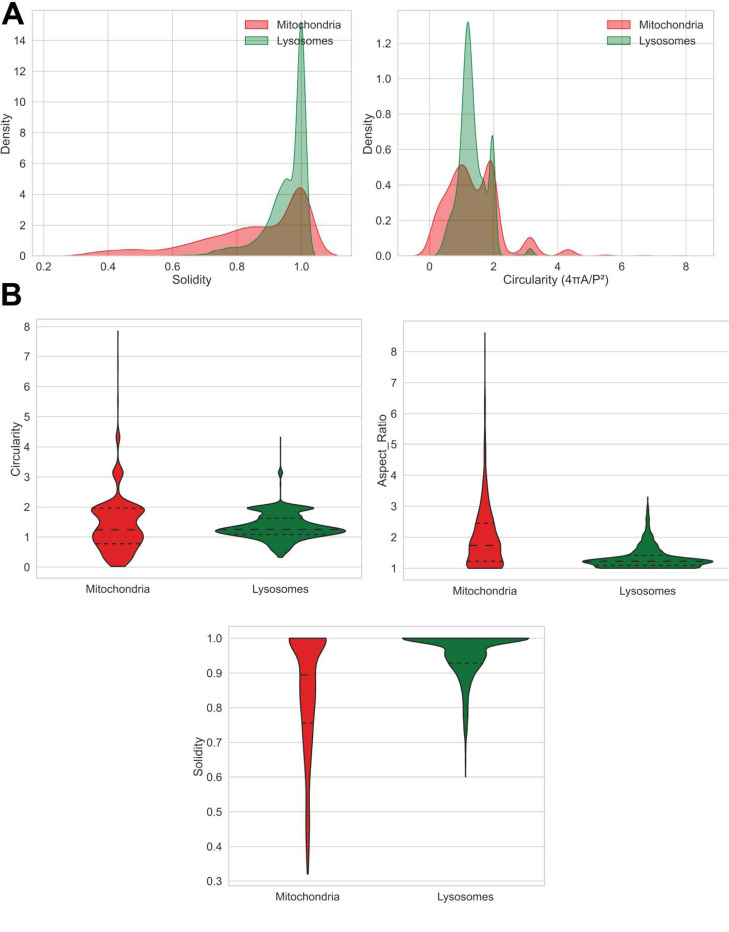
Quantitative shape profiling of organelles. (**A**) Continuous shape descriptors extracted for all detected organelles are summarized as parameter distributions showing variability in area, circularity, solidity, eccentricity, and aspect ratio within a single neuron. (**B**) Comparative violin plots illustrate differences in these shape metrics between mitochondria and lysosomes, providing a continuous morphological quantification beyond categorical classification.

Beyond binary classification, AutoMorphoTrack extracts shape descriptors for each segmented organelle. Distributions of parameters such as circularity, solidity, eccentricity, aspect ratio, and area summarize the range of morphological states of organelles (Figure 2A). Violin plots comparing the same descriptors for mitochondria and lysosomes (Figure 2B) provide a continuous morphological profile that complements prior classification metrics.

### Organelle Tracking and Motility Analysis

**Figure 3.**
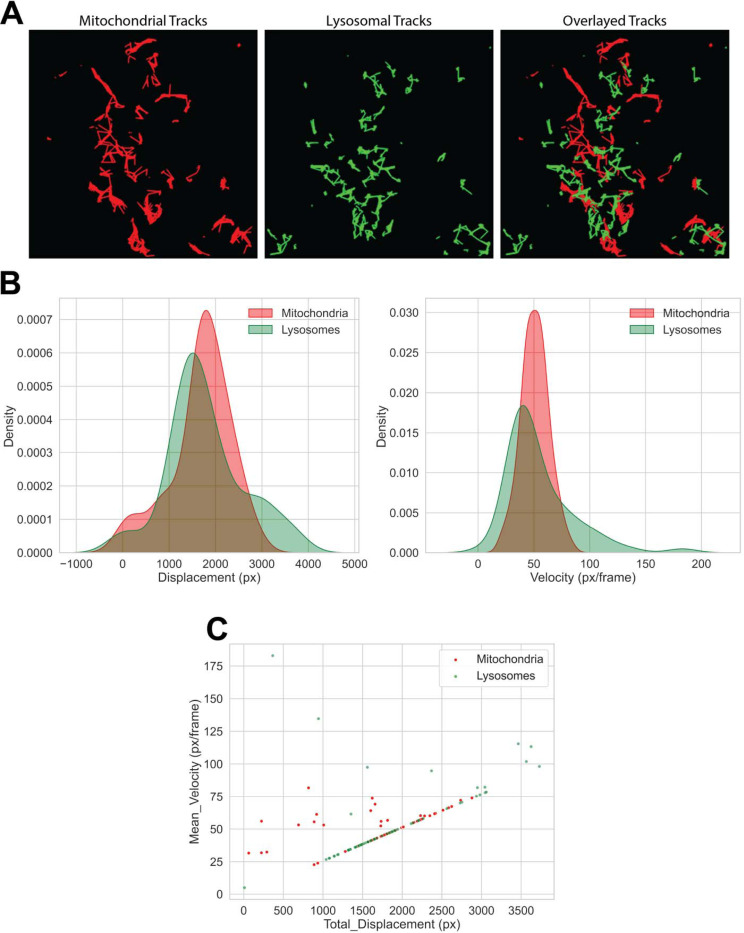
Organelle tracking and motility characterization. (**A**) Centroid-based trajectory reconstructions show cumulative lysosomal and mitochondrial movements and a composite overlay of both populations. (**B**) Quantitative motility metrics are summarized as velocity and displacement distributions. (**C**) A scatter plot of individual trajectories, together capturing the dynamic range of organelle transport within a neuron.

Centroid-based nearest-neighbor linkage reconstructs organelle paths across frames. Cumulative-track overlays show the independent motion of lysosomes, mitochondria, and their composite trajectories when both populations are visualized together (Figure 3A). Corresponding velocity and displacement distributions (Figure 3B) depict organelle motility characteristics, while a scatter plot (Figure 3C) summarizes individual organelle trajectories. Together, these panels quantify the dynamic behavior of organelles within single neurons.

### Colocalization and Interaction Metrics

**Figure 4.**
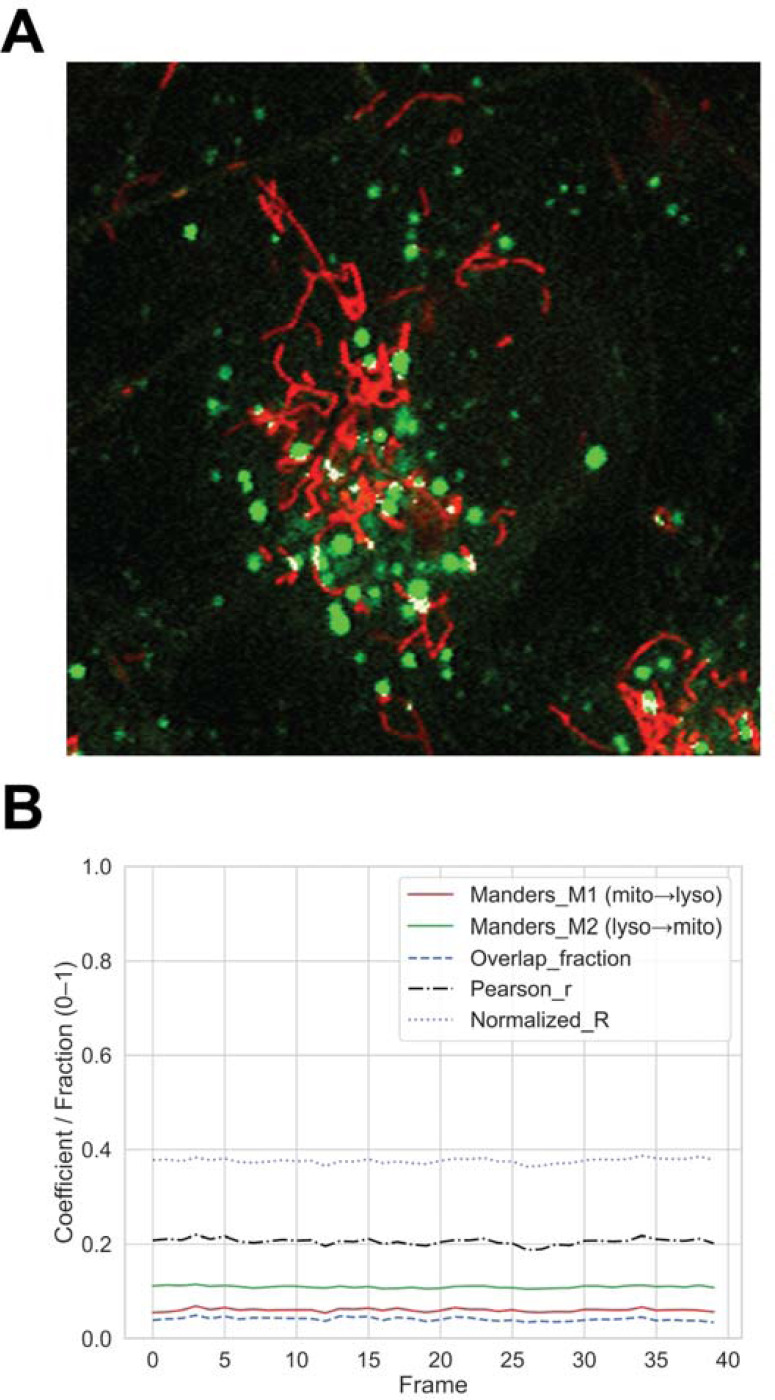
Mitochondria–lysosome colocalization within single neurons. (**A**) Representative overlay highlighting regions of spatial overlap between mitochondria and lysosomes. (**B**) Quantitative summary plot showing Manders M1/M2 and Pearson *r* coefficients across time. These analyses quantify the frequency and degree of inter-organelle contact within the cell.

Spatial overlap between mitochondria and lysosomes is quantified using both Manders’ and Pearson’s coefficients. A representative overlay highlights regions of direct spatial proximity (Figure 4A), while a metrics plot summarizes Manders M1/M2 and Pearson *r* coefficients across time (Figure 4B). These results capture the extent and stability of mitochondria–lysosome interactions within individual neurons, across the timeframe in which the cell is observed.

### Integrated Correlation Summary

**Figure 5.**
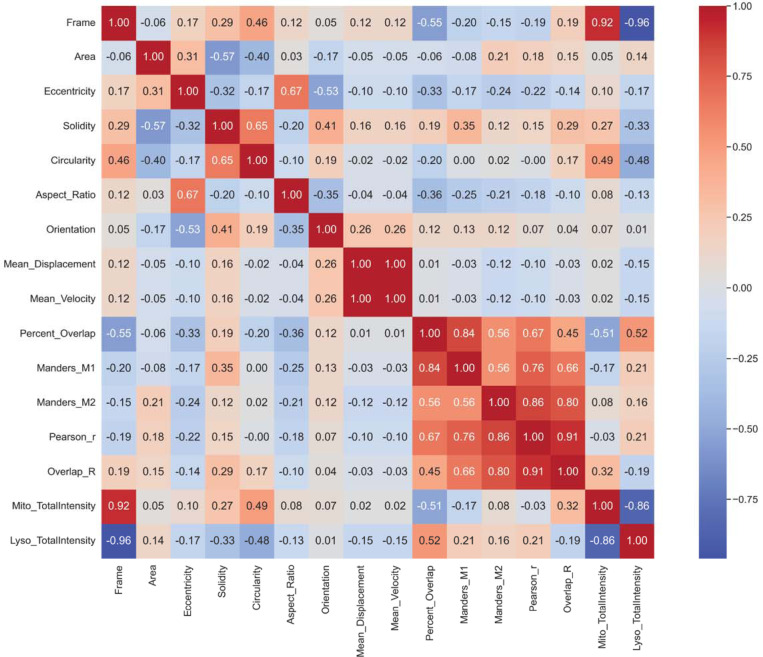
Integrated correlation of morphology, motility, and colocalization metrics. Comprehensive correlation matrix displaying pairwise Pearson relationships among morphological descriptors, motility parameters, and colocalization indices derived from AutoMorphoTrack outputs. The matrix reveals coordinated trends linking structural and dynamic features within a single neuron.

To assess how structural and functional properties interrelate, AutoMorphoTrack merges all outputs into an integrated dataset. A single correlation matrix (Figure 5) illustrates the pairwise relationships between morphological descriptors, motility parameters, and colocalization indices, providing a multidimensional representation of organelle behavior within one neuron.

### Cross-Neuron Comparative Analysis

Two different image stacks were analyzed in parallel to demonstrate the reproducibility and extensibility of AutoMorphoTrack outputs.

**Figure 6.**
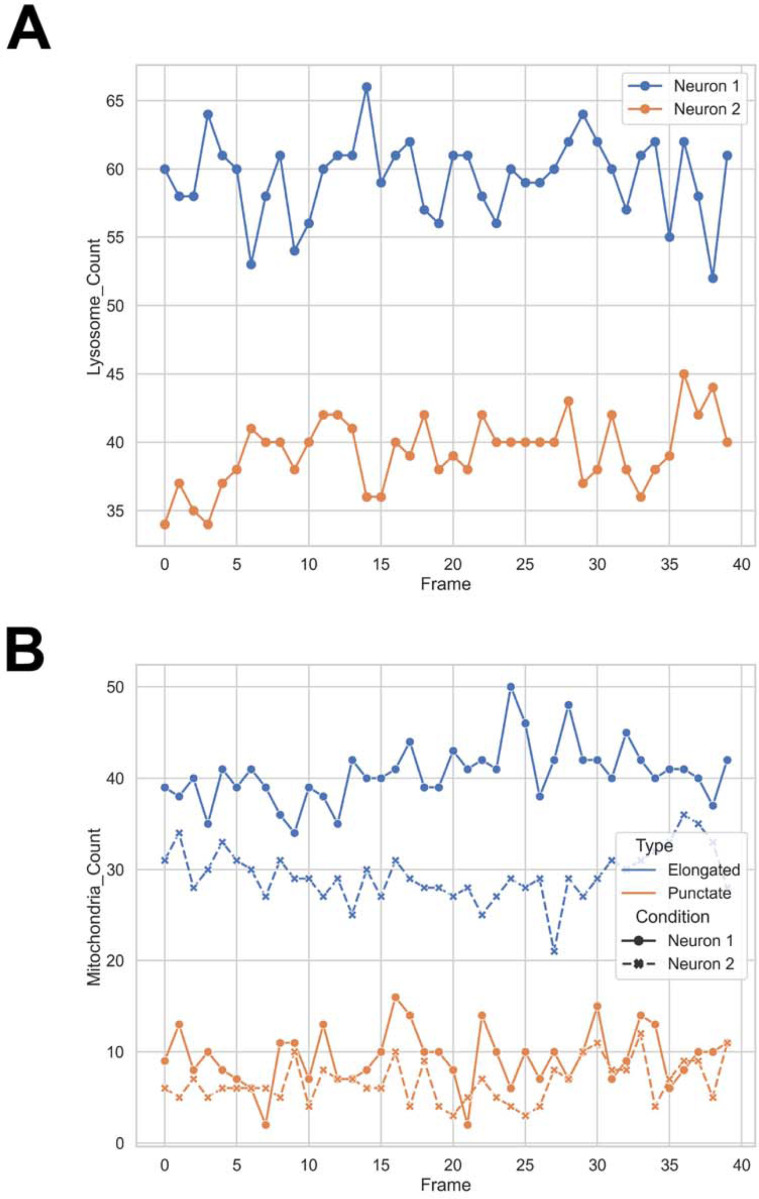
Lysosome and mitochondrial comparisons between neurons. (**A**) Comparative plots showing differences in lysosomal abundance per frame and (**B**) mitochondrial morphology classification between two neurons. Variations highlight cell-specific differences in vesicle turnover and mitochondrial network organization.

Figure 6 compares lysosomal abundance (Figure 6A) and mitochondrial morphology profiles (Figure 6B) between the two neurons, revealing clear cell-to-cell variability in lysosomal turnover and mitochondrial morphology.

**Figure 7.**
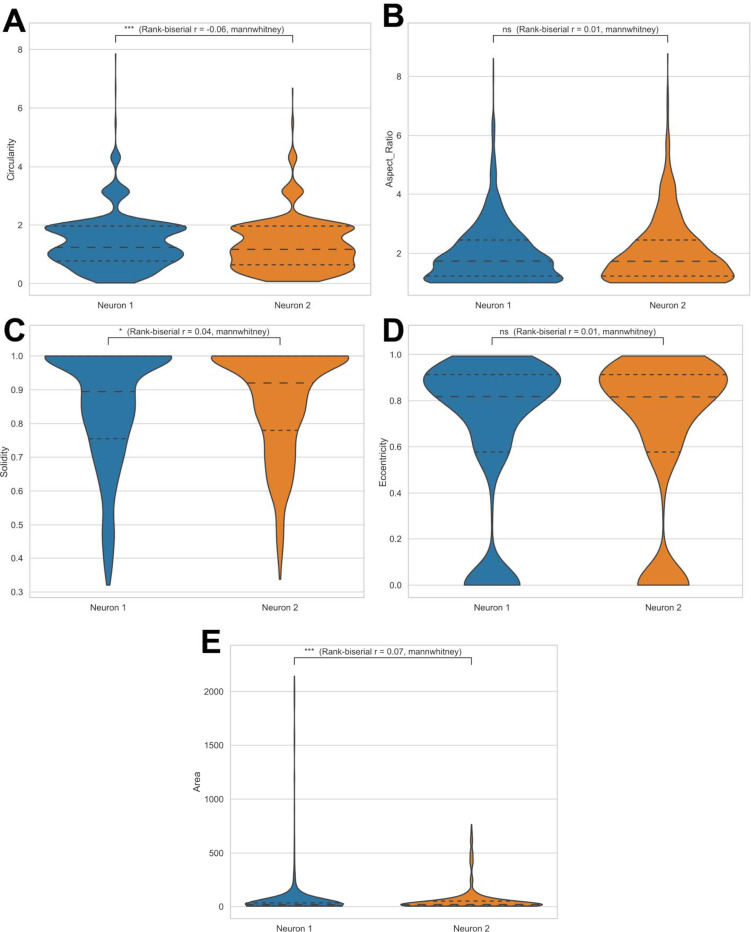
Comparative shape metrics across neurons. Violin plots comparing (**A**) circularity, (**B**) aspect ratio, (**C**) solidity, (**D**) eccentricity, and (**E**) area between Neuron 1 and Neuron 2. Distinct parameter distributions reveal structural heterogeneity and variability in organelle geometry across cells.

Figure 7 summarizes differences in shape metrics, showing violin plots for area (Figure 7A), circularity (Figure 7B), solidity (Figure 7C), eccentricity (Figure 7D), and aspect ratio (Figure 7E). These comparisons highlight distinct geometric characteristics that distinguish the organelles across the two image stacks.

**Figure 8.**
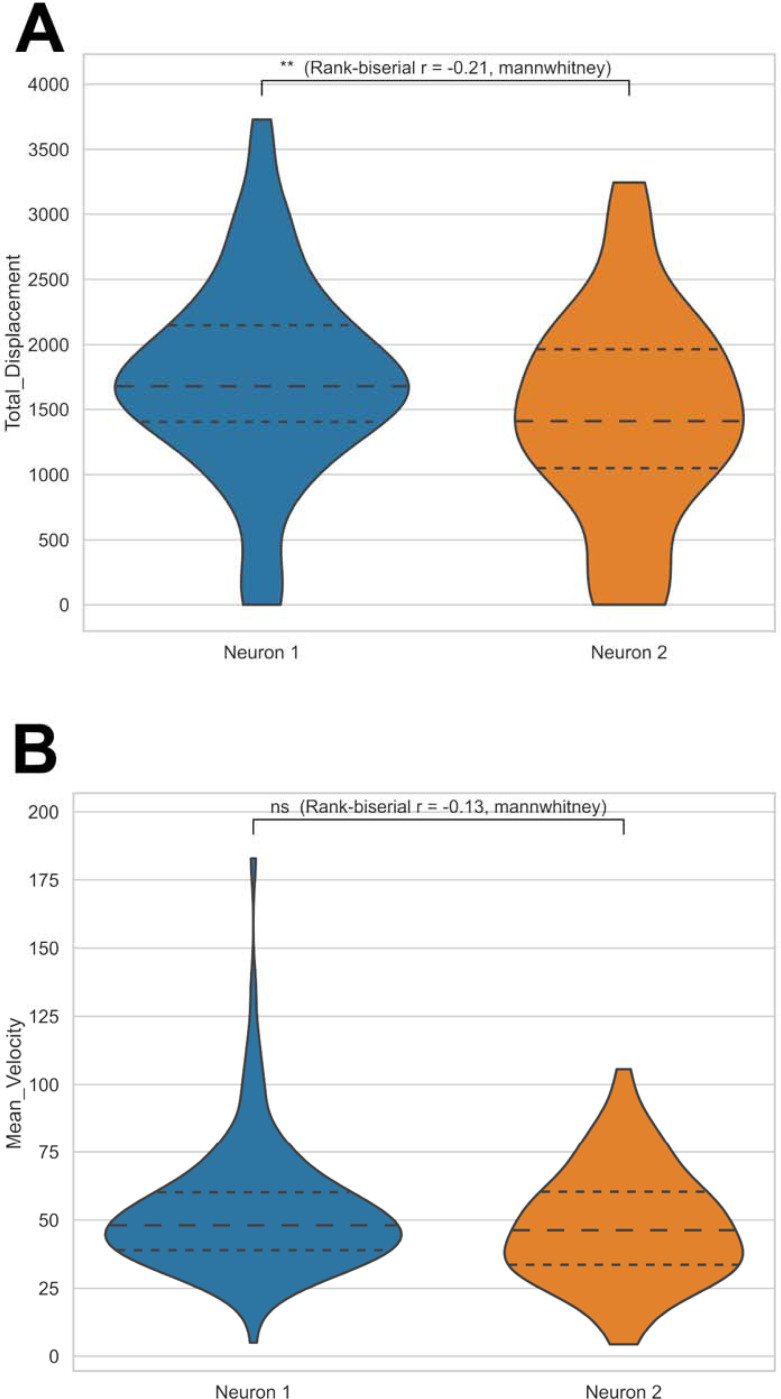
Comparative motility parameters between neurons. Distributions of total displacement (**A**) and mean velocity (**B**) for mitochondria and lysosomes from two neurons. Differences demonstrate cell-specific organelle transport dynamics and variability in overall motility behavior.

Motility differences are illustrated in Figure 8, where mean velocity (Figure 8A) and total displacement (Figure 8B) distributions reveal neuron-specific patterns of organelle movement.

**Figure 9.**
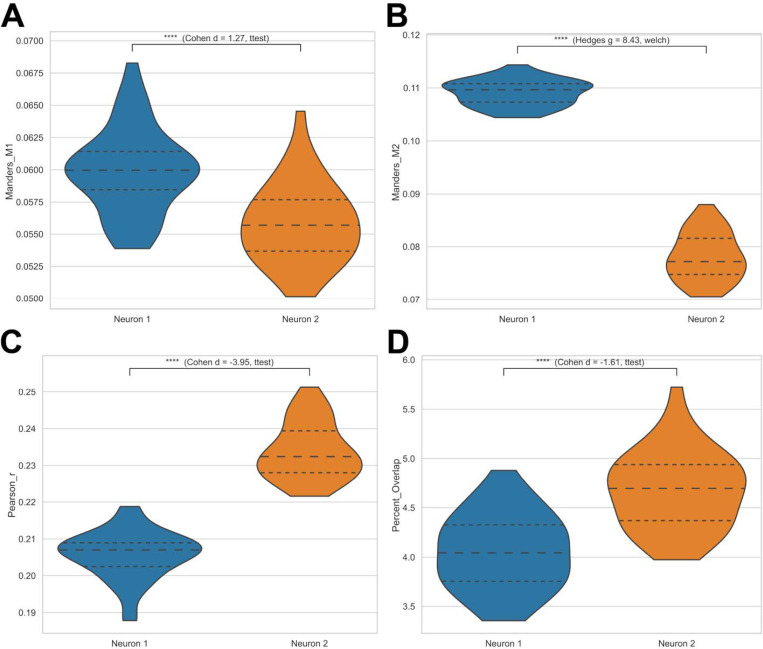
Comparative colocalization metrics between neurons. Violin plots showing differences in Manders M1 (**A**), Manders M2 (**B**), Pearson *r* (**C**), and percent overlap (**D**) between Neuron 1 and Neuron 2. Divergent distributions indicate variability in mitochondria–lysosome coupling efficiency across cells.

Variability in inter-organelle coupling/colocalization is shown in Figure 9, which presents comparative analyses of Manders M1 (Figure 9A), Manders M2 (Figure 9B), Pearson *r* (Figure 9C), and percent overlap (Figure 9D) between neurons. These comparisons demonstrate distinct levels of mitochondria–lysosome colocalization and coordination.

**Figure 10.**
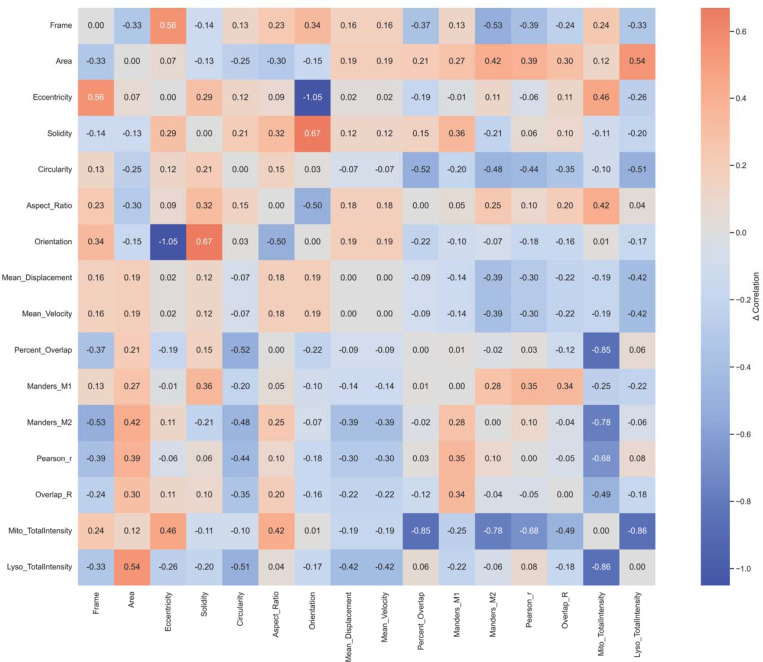
Differential correlation matrix between neurons. Subtraction map of correlation matrices from Neuron 1 and Neuron 2, highlighting parameters whose inter-relationships differ most strongly between cells. The heatmap summarizes shifts in coordination among morphology, motility, and interaction features, revealing neuron-specific patterns of subcellular organization.

Finally, Figure 10 visualizes the difference between the two neurons’ integrated correlation matrices, highlighting parameters whose relationships shift most strongly between cells.

## Discussion

This study introduces AutoMorphoTrack, an automated, open-source pipeline for quantitative analysis of organelle morphology, motility, and spatial interactions. By combining transparent modular design with reproducible data handling, AutoMorphoTrack bridges a major gap between high-content microscopy and quantitative cell biology. Previous image analysis tools, including ImageJ plugins such as LIM Tracker and MTrack, have enabled segmentation or tracking of subcellular structures, but each typically focuses on a single task or organelle class ^7,8^. AutoMorphoTrack integrates and extends these capabilities within a unified, reproducible workflow. The toolkit enables non-programming users to perform sophisticated image analyses that were previously restricted to computational specialists.

The analyses performed here demonstrate that AutoMorphoTrack accurately segments and tracks subcellular structures while preserving biologically relevant variability. Within single image stacks (featuring single neurons), the pipeline captured continuous morphological diversity and revealed distinct populations of elongated and punctate mitochondria. Such heterogeneity reflects the balance between mitochondrial fusion and fission, processes that maintain organelle quality control and metabolic adaptability. Similarly, lysosomal counts and motility trajectories varied dynamically within individual cells, consistent with the notion that lysosomal positioning is actively regulated by microtubule transport and local metabolic cues. The ability of AutoMorphoTrack to simultaneously visualize and quantify these events allows the identification of subtle dynamic features that would otherwise remain qualitative.

When applied to two independent image stacks, AutoMorphoTrack revealed substantial cell-to-cell differences in every major dimension of organelle organization. Morphology-based comparisons indicated that the overall distribution of mitochondrial shapes differs between neurons, suggesting variability in network connectivity, mitochondrial dynamics, or energetic demand. Shape-descriptor comparisons further showed that structural parameters such as circularity, solidity, eccentricity, and aspect ratio diverge across cells, emphasizing that even morphologically similar neurons maintain unique subcellular geometries. These differences are not artifacts of imaging or segmentation but reflect genuine biological heterogeneity at the single-cell level, as all these variabilities are controlled for through consistent thresholding and size parameters in AutoMorphoTrack.

Motility analyses uncovered additional layers of diversity. The distributions of mean velocity and total displacement indicated that one neuron harbored more mobile organelles than the other, pointing to differences in cytoskeletal organization or intracellular trafficking. When comparing neurons undergoing different experimental treatments, these changes can provide mechanistic insights as to minute changes caused by the experimental variable. The inclusion of colocalization analyses further extended these insights by quantifying mitochondria–lysosome interactions, a critical component of organelle quality control. Divergent values in Manders’ coefficients and Pearson correlations between neurons suggest cell-specific differences that can be further adapted to neurons captured in different experimental conditions.

Beyond its biological insights, AutoMorphoTrack offers a methodological advance in how image-derived data are structured and analyzed. Each processing step produces standardized outputs that can be directly used for downstream statistical analysis or integration within other computational frameworks. The optional comparison script, AMTComparison.py, exemplifies this extension. By remaining external to the core package, it allows users to adopt or modify statistical approaches suited to their experimental design while maintaining full reproducibility.

Overall, AutoMorphoTrack transforms subcellular imaging into a quantitative, reproducible, and scalable workflow that connects structural, kinetic, and interaction data within single cells. It not only enables the identification of subtle differences in organelle morphology and dynamics but also provides a reproducible framework for comparing these parameters across individual conditions or treatments. By merging accessibility with analytical depth, AutoMorphoTrack advances the study of organelle behavior from descriptive observation to quantitative, statistically rigorous insight.

## Conclusion

AutoMorphoTrack provides an end-to-end solution for quantitative, reproducible, and accessible analysis of organelle morphology, motility, and spatial interactions. By unifying segmentation, tracking, and correlation analysis within a single open-source framework, the toolkit enables consistent, high-content evaluation of subcellular organization across imaging modalities and experimental designs. The inclusion of standardized outputs and an adaptable comparison workflow allows users to extend the analysis toward inter-neuron or inter-condition comparisons without modifying the underlying code.

Through its modular design, AutoMorphoTrack can be readily adapted to any pair of fluorescently labeled organelles, extending its relevance beyond mitochondria and lysosomes to other compartments such as endosomes, peroxisomes, or autophagosomes. The capacity to quantify and compare these parameters across individual cells or conditions lays the groundwork for studying how subcellular coordination varies in health and disease. As imaging datasets continue to expand in complexity, AutoMorphoTrack provides a transparent and reproducible foundation for bridging cell biology with computational image analysis—transforming qualitative microscopy into a quantitative framework for mechanistic discovery.

## Supplementary Material

Supplement 1

Supplement 2

Supplement 3

Supplement 4

## Figures and Tables

**Table 1.** Functional Steps in the AutoMorphoTrack Workflow

Step	Function	Key User Parameters	Biological Insight
1	Organelle segmentation	thr_factor, min_size	Detect mitochondria and lysosomes
2	Lysosome enumeration	fps, thr_factor	Temporal vesicle dynamics
3	Morphology classification	area_thresh, ecc_thresh	Network fragmentation vs elongation
4	Shape metric extraction	min_size, thr_factor	Continuous structural profiling
5	Trajectory tracking	max_dist, fps	Organelle transport behavior
6	Colocalization analysis	mask_sensitivity, threshold	Mitochondria–lysosome interactions
7	Integrated summary	merge_mode, corr_method	Correlative morphology-motility metrics
8	Comparative analysis (optional)	Statistical test selection	Cross-sample variability

**Table 2.** AutoMorphoTrack Package Structure

Module	Core Function	Input	Primary Output
detection.py	Segment mitochondria and lysosomes	Multichannel.tif	Binary masks + overlays
lyso_count.py	Count lysosomes across frames	Channel mask	Counts per frame + plot
morphology.py	Classify mitochondrial shapes	Mito mask	Labeled images + summary table
shape_features.py	Extract quantitative shape descriptors	Binary masks	Shape metrics tables
tracking.py	Reconstruct organelle trajectories	Mask sequence	Tracks + cumulative overlays
motility.py	Compute displacement and velocity	Track coordinates	Motility summaries
colocalization.py	Measure overlap and Pearson/Manders indices	Binary masks	Colocalization metrics
summary.py	Integrate and correlate metrics	All outputs	Correlation heatmaps
utils.py	Shared helper functions	Global	Directory and format management
AMTComparison.py	(Optional) Statistical comparison	CSV outputs	Violin plots + effect sizes

**Table 3.** Representative Outputs Generated by AutoMorphoTrack

Step	Primary Outputs	Output Type	Purpose
1	Mito_Frame0.png, Lyso_Frame0.png, *_Detection.mp4	Image + Video	Visualize segmented organelles
2	Lyso_Frame0_Count.png, Lysosome_Counts.csv, Lyso_Count_Plot.png	Image + CSV	Quantify lysosome abundance
3	Morphology_Frame0_Labeled.png, Morphology_Summary.csv	Image + CSV	Classify mitochondrial networks
4	Mito_ShapeMetrics.csv, Lyso_ShapeMetrics.csv, Shape_ViolinPlots.png	CSV + Plot	Profile organelle shape metrics
5	Cumulative_Mito.png, Mito_Tracks.csv, Motility_Scatter.png	Image + CSV	Quantify motility parameters
6	Colocalization_Frame0.png, Colocalization.csv, Colocalization_MetricsPlot.png	Image + CSV	Assess organelle overlap
7	Integrated_CorrelationMatrix.png, Integrated_Merged_Data.csv	Plot + CSV	Summarize interparameter relationships
8	*_Comparison.png, *_Comparison.csv	Plot + CSV	Compare neurons or conditions (optional)

## Data Availability

Outputs generated using AutoMorphoTrack can be found at: Bayati, Armin (2025), “AutoMorphoTrack: A modular framework for quantitative analysis of organelle morphology, motility, and interactions at single-cell resolution”, Mendeley Data, V1, doi: 10.17632/hjnzjcfwjm.1 Package can be accessed from: https://github.com/abayatibrain/AMTpackage.git Jupyter Notebook version of this code can be accessed from: https://github.com/abayatibrain/automorphotrack.git Comparison code, used to compare outputs of AutoMorphoTrack generated from analyzing different image stacks can be found at: https://github.com/abayatibrain/AMTcomparison.git
