# Supplementary figures and images for: AutoMorphoTrack: A modular framework for quantitative analysis of organelle morphology, motility, and interactions at single-cell resolution
Source: bioRxiv. 2026 Feb 2:2025.07.19.665650. Preprint. [Version 5] doi: 10.1101/2025.07.19.665650 (PMC12889450; doi:10.1101/2025.07.19.665650)

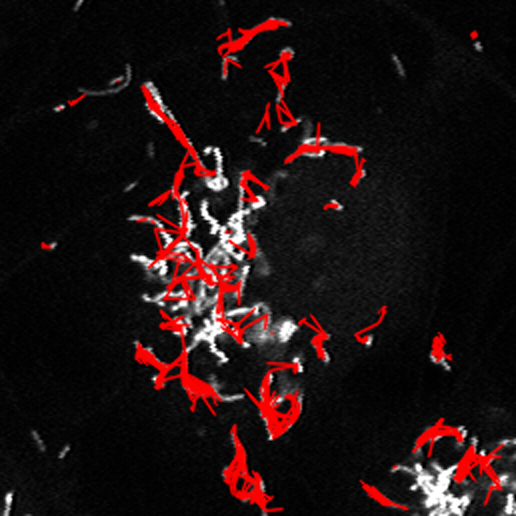

Supplement: Supplement 1 [file media-1.zip › Composite8/Step4_5_Tracking_Outputs/Step4.5_Cumulative_Mito.png]

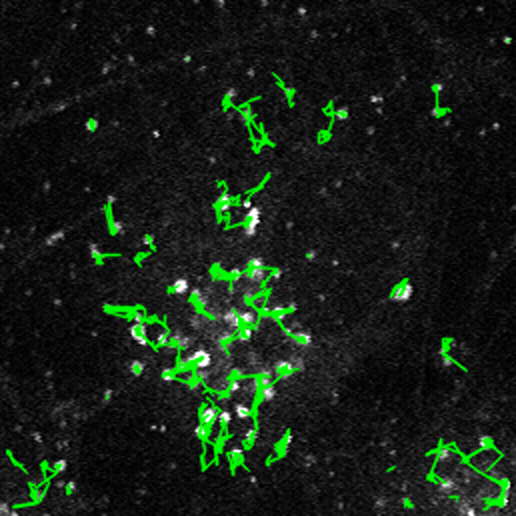

Supplement: Supplement 1 [file media-1.zip › Composite8/Step4_5_Tracking_Outputs/Step4.5_Cumulative_Lyso.png]

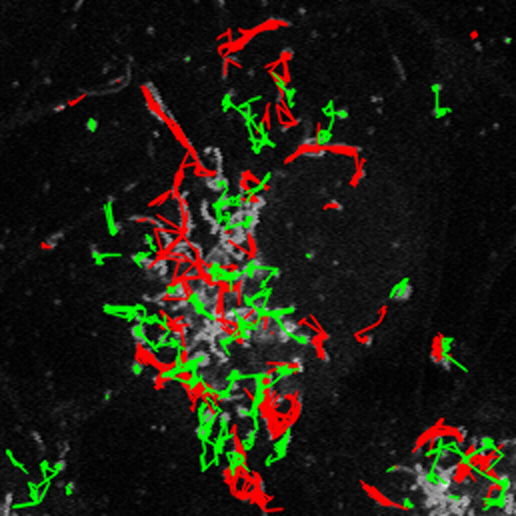

Supplement: Supplement 1 [file media-1.zip › Composite8/Step4_5_Tracking_Outputs/Step4.5_Cumulative_Composite.png]

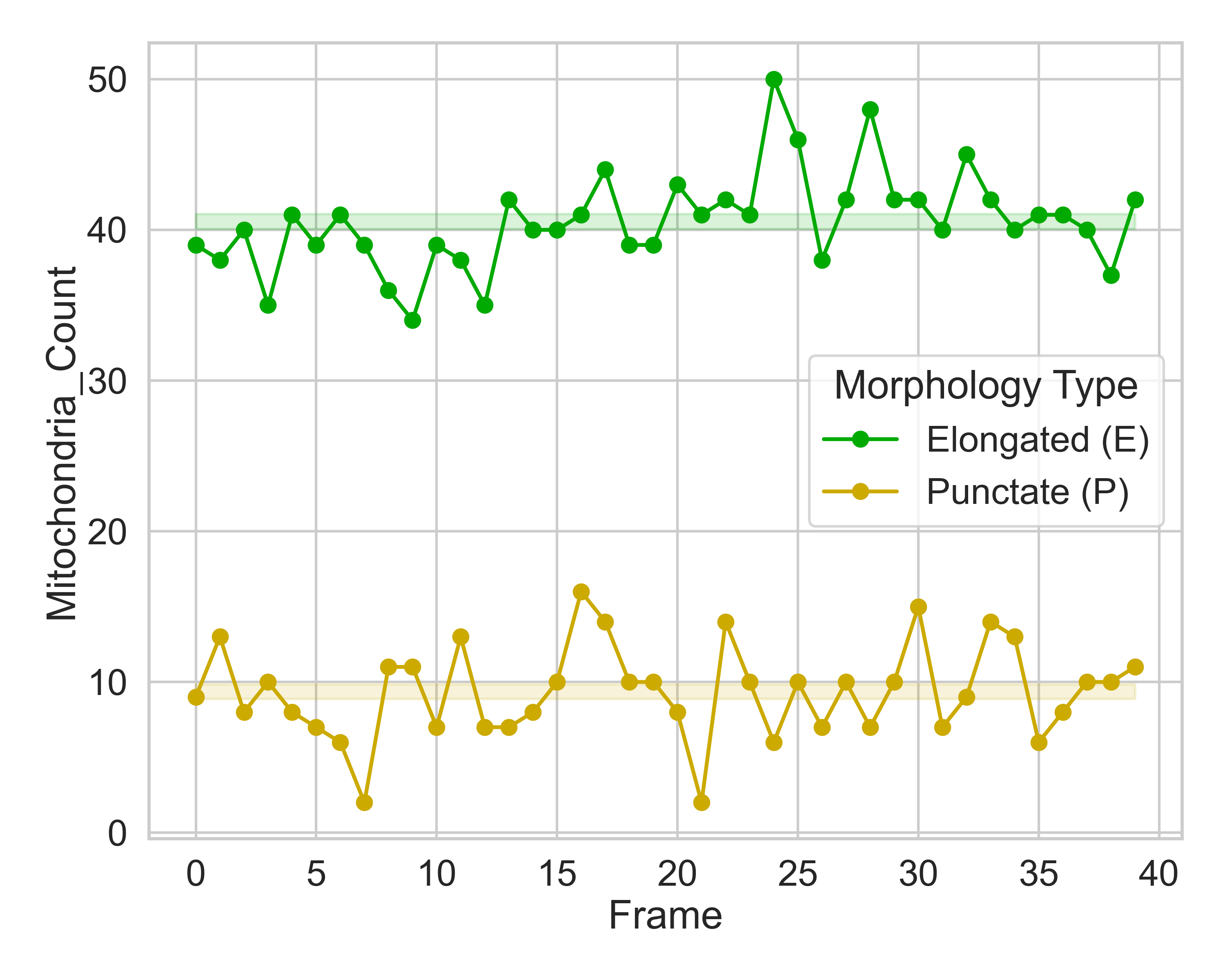

Supplement: Supplement 1 [file media-1.zip › Composite8/Step2_Morphology_Outputs/Step2_Morphology_AcrossFrames.png]

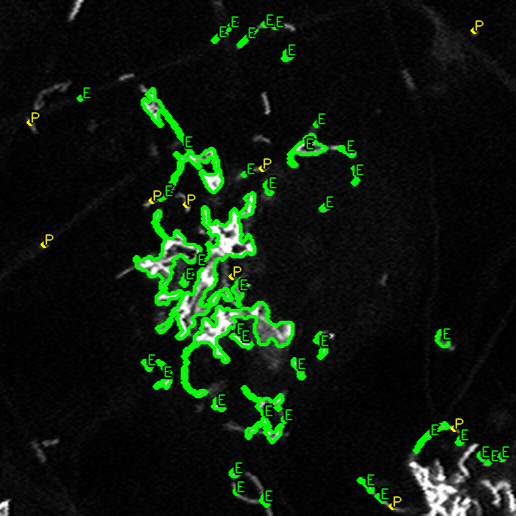

Supplement: Supplement 1 [file media-1.zip › Composite8/Step2_Morphology_Outputs/Step2_Frame0_Labeled.png]

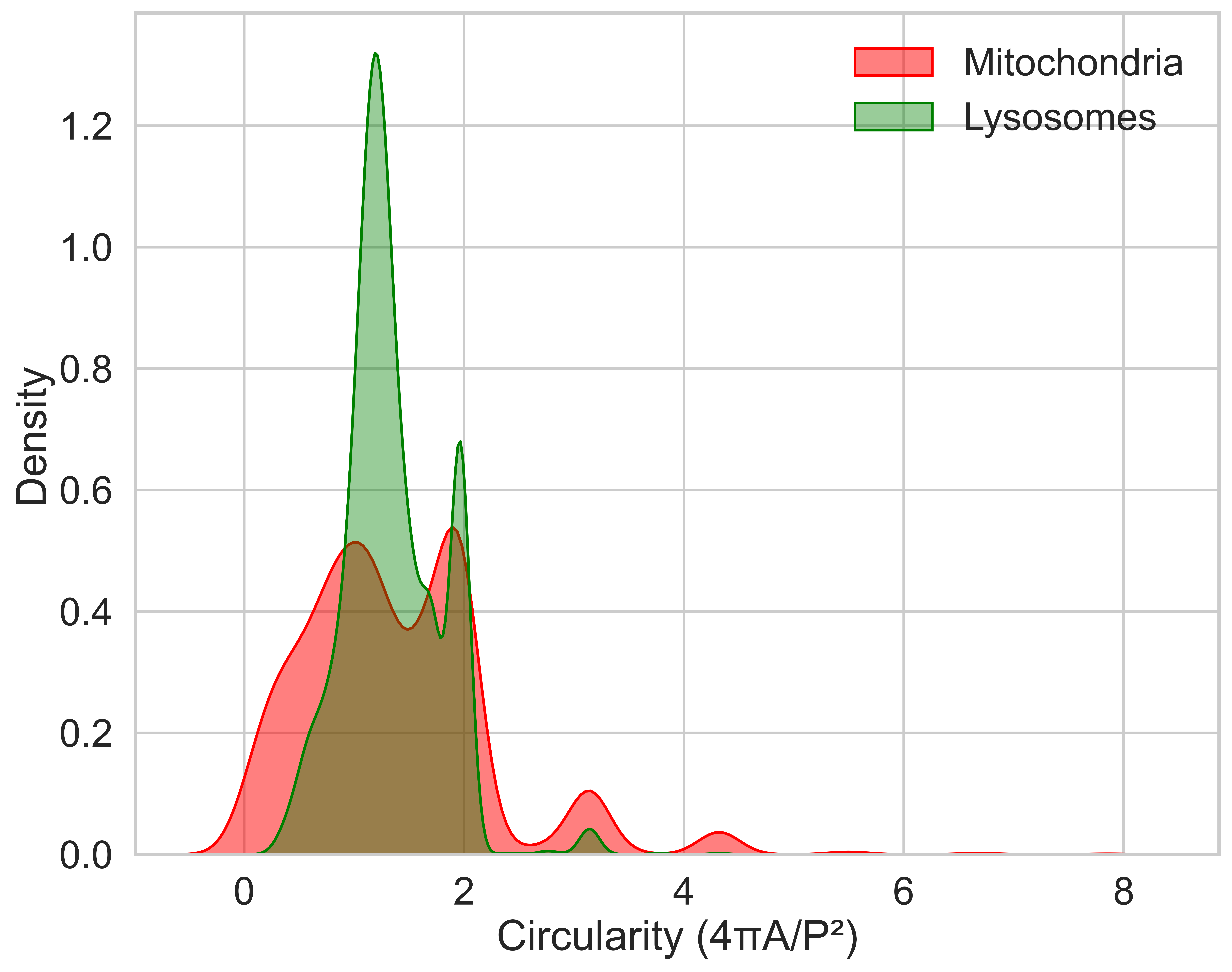

Supplement: Supplement 1 [file media-1.zip › Composite8/Step3_Shape_Analysis_Outputs/Step3_Circularity_Distribution.png]

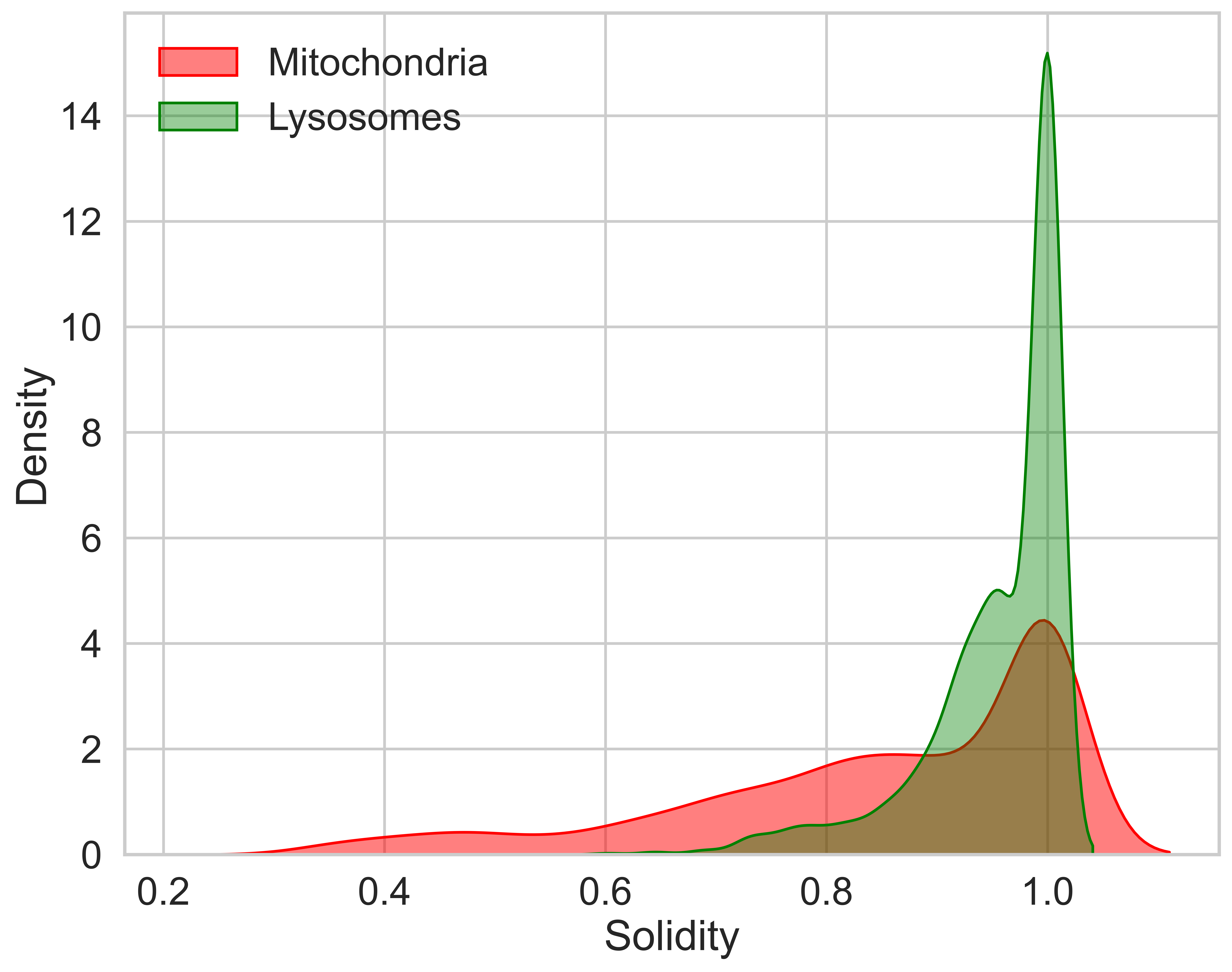

Supplement: Supplement 1 [file media-1.zip › Composite8/Step3_Shape_Analysis_Outputs/Step3_Solidity_Distribution.png]

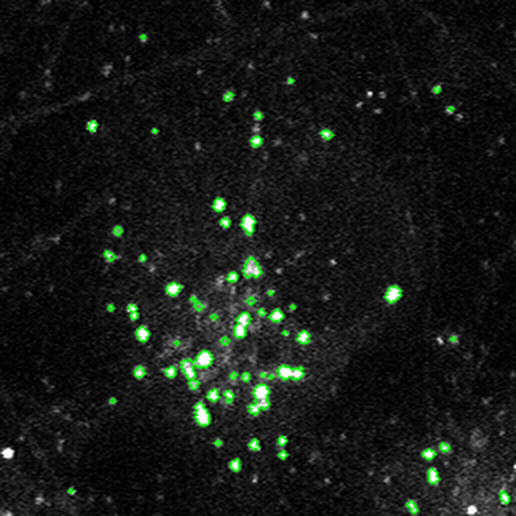

Supplement: Supplement 1 [file media-1.zip › Composite8/Step1_Detection_Outputs/Step1_Lyso_Frame0.png]

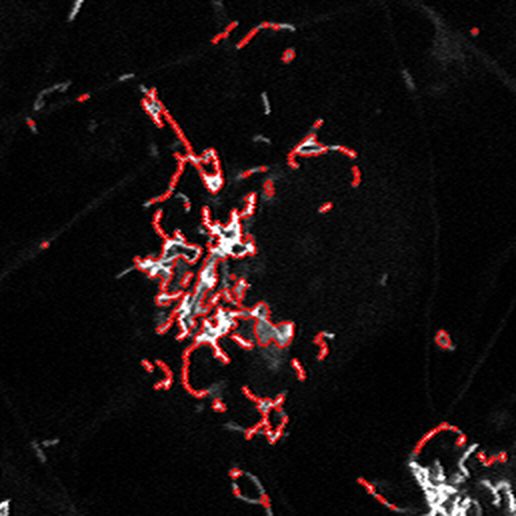

Supplement: Supplement 1 [file media-1.zip › Composite8/Step1_Detection_Outputs/Step1_Mito_Frame0.png]

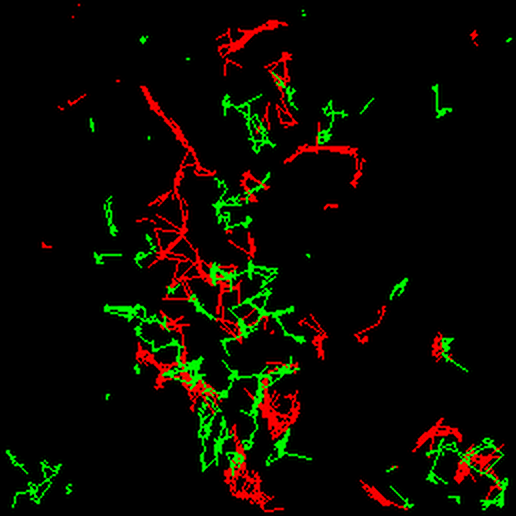

Supplement: Supplement 1 [file media-1.zip › Composite8/Step4_Tracking_Outputs/Step4_Cumulative_Composite.png]

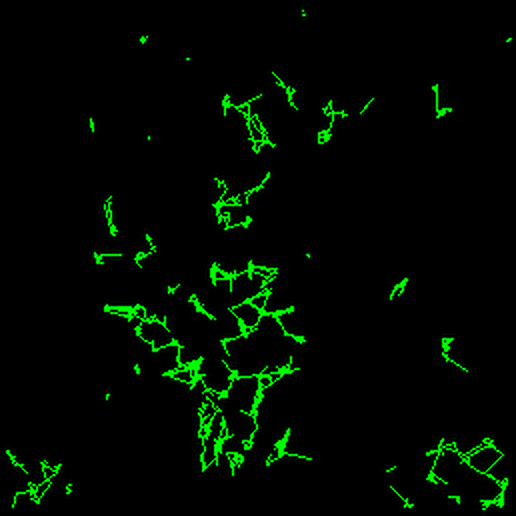

Supplement: Supplement 1 [file media-1.zip › Composite8/Step4_Tracking_Outputs/Step4_Cumulative_Lyso.png]

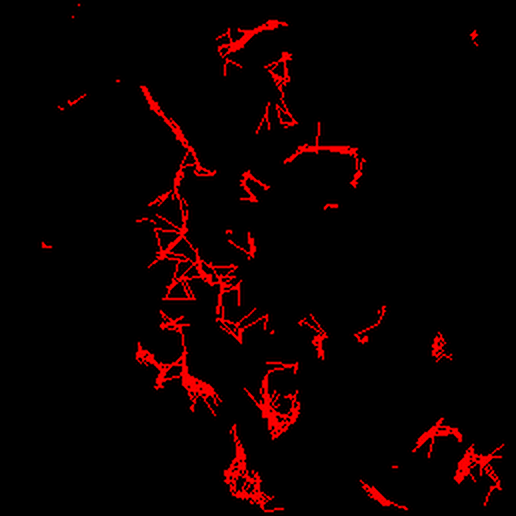

Supplement: Supplement 1 [file media-1.zip › Composite8/Step4_Tracking_Outputs/Step4_Cumulative_Mito.png]

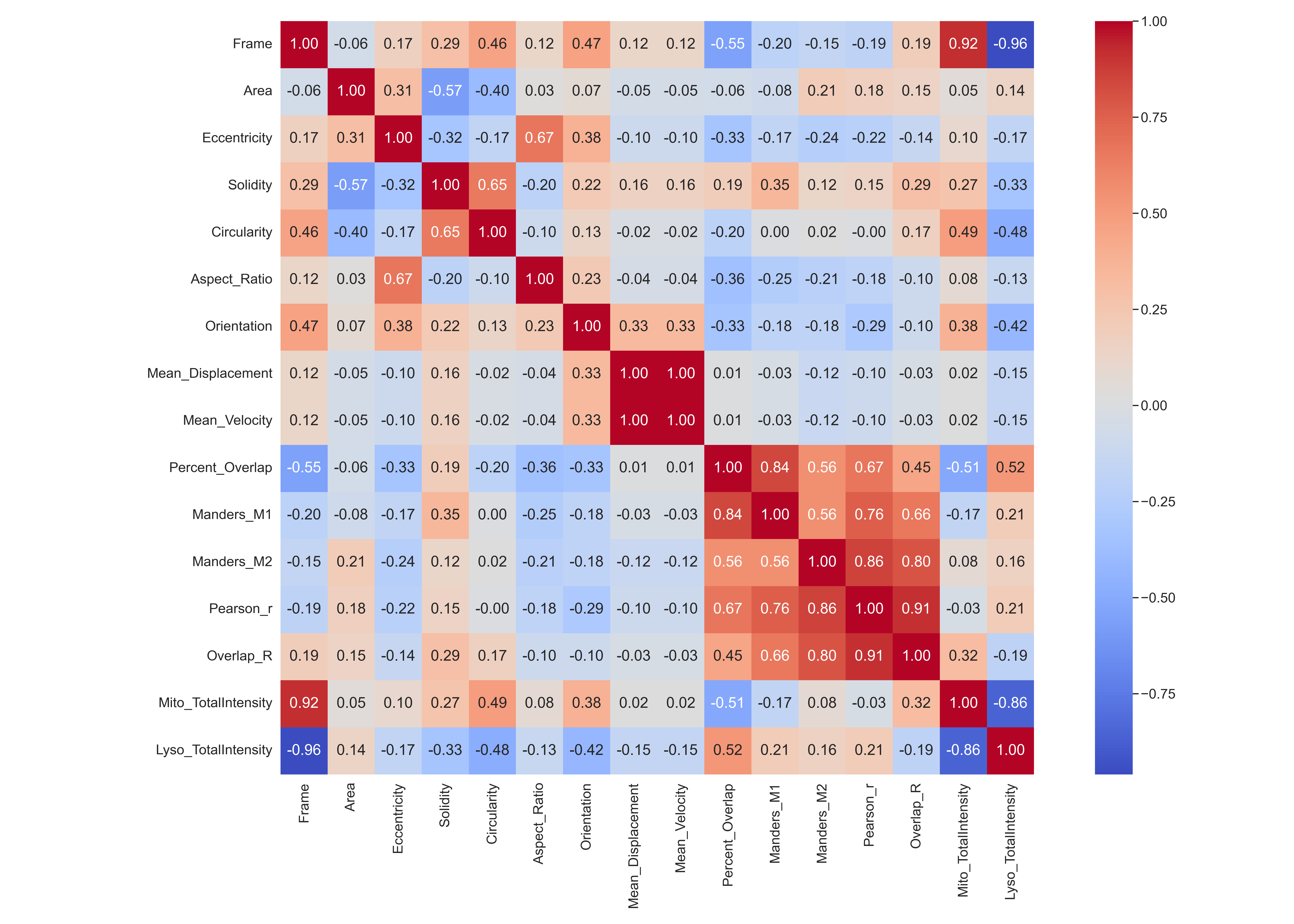

Supplement: Supplement 1 [file media-1.zip › Composite8/Step7_Integrated_Summary_Outputs/Step7_Integrated_CorrelationMatrix.png]

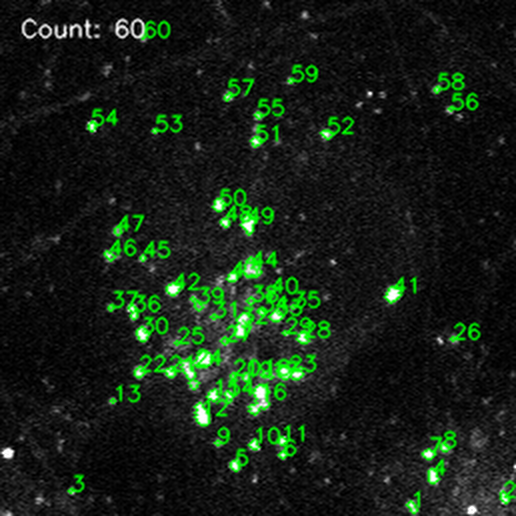

Supplement: Supplement 1 [file media-1.zip › Composite8/Step1_Lyso_Count_Outputs/Lysosomes_Frame0_with_Count.png]

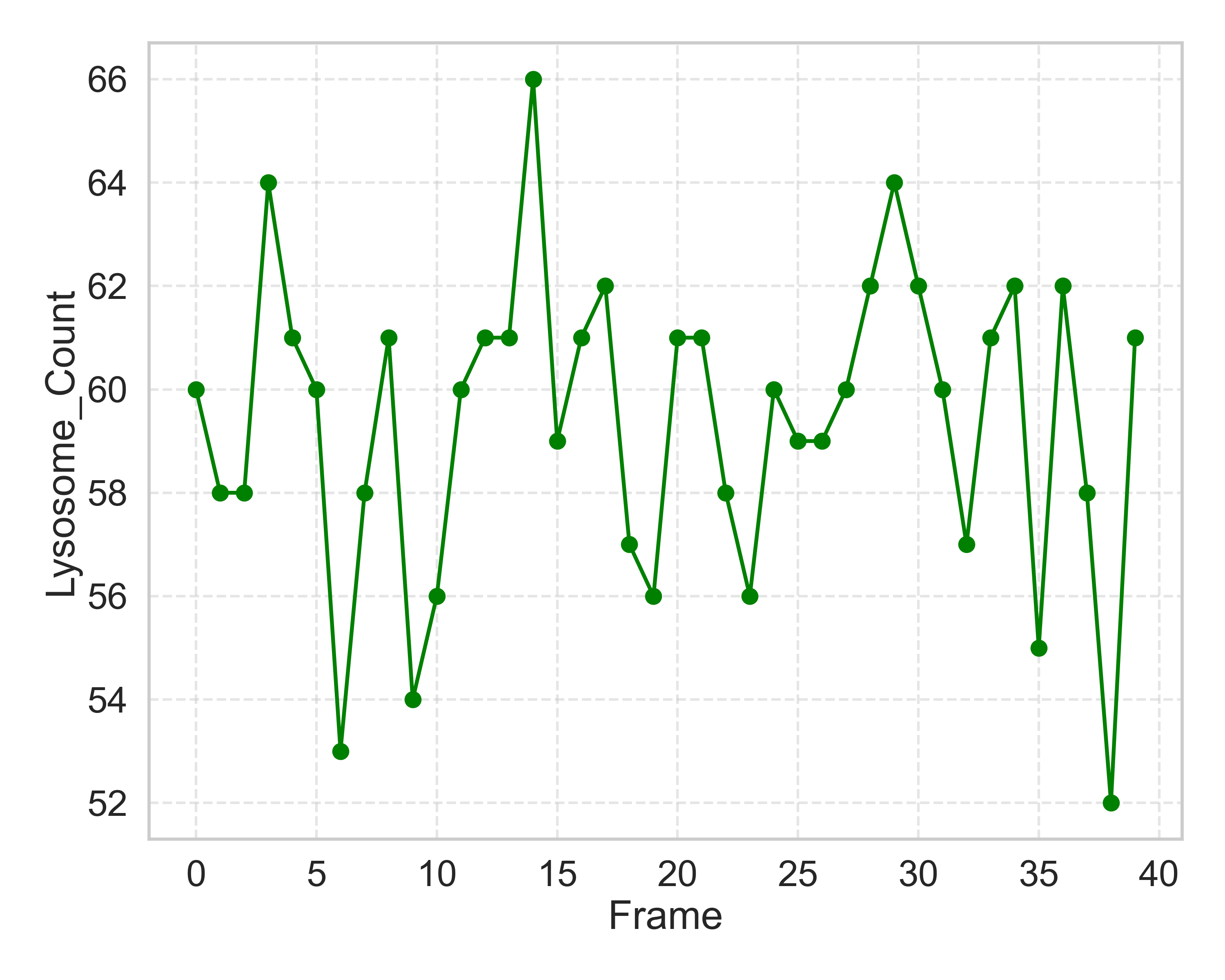

Supplement: Supplement 1 [file media-1.zip › Composite8/Step1_Lyso_Count_Outputs/Lysosome_Count_Plot.png]

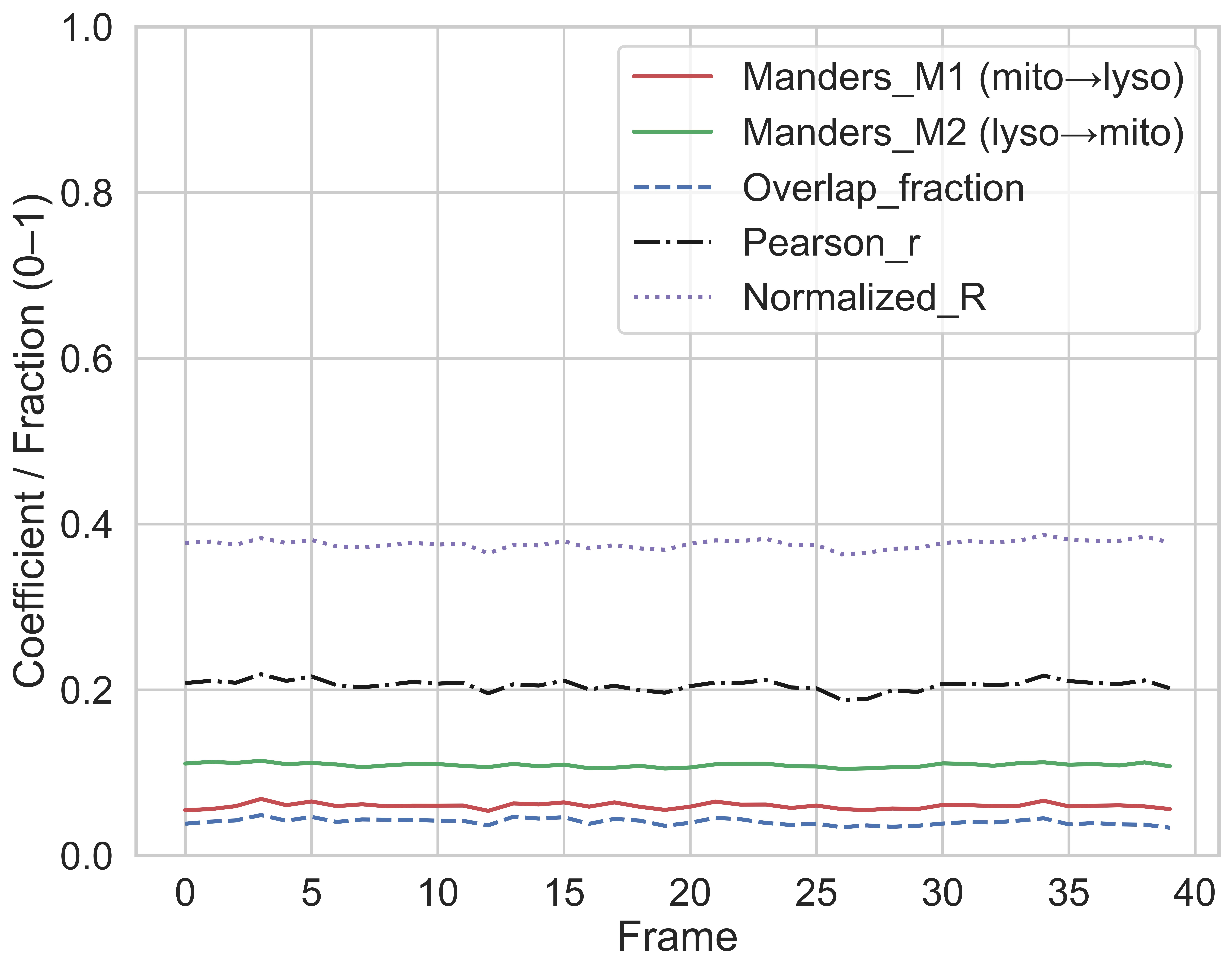

Supplement: Supplement 1 [file media-1.zip › Composite8/Step6_Colocalization_Outputs/Step6_HQ_AllMetricsPlot.png]

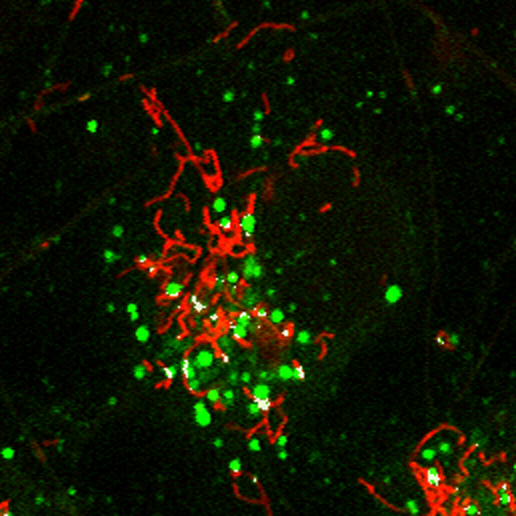

Supplement: Supplement 1 [file media-1.zip › Composite8/Step6_Colocalization_Outputs/Step6_HQ_Frame0_Overlay_white.png]

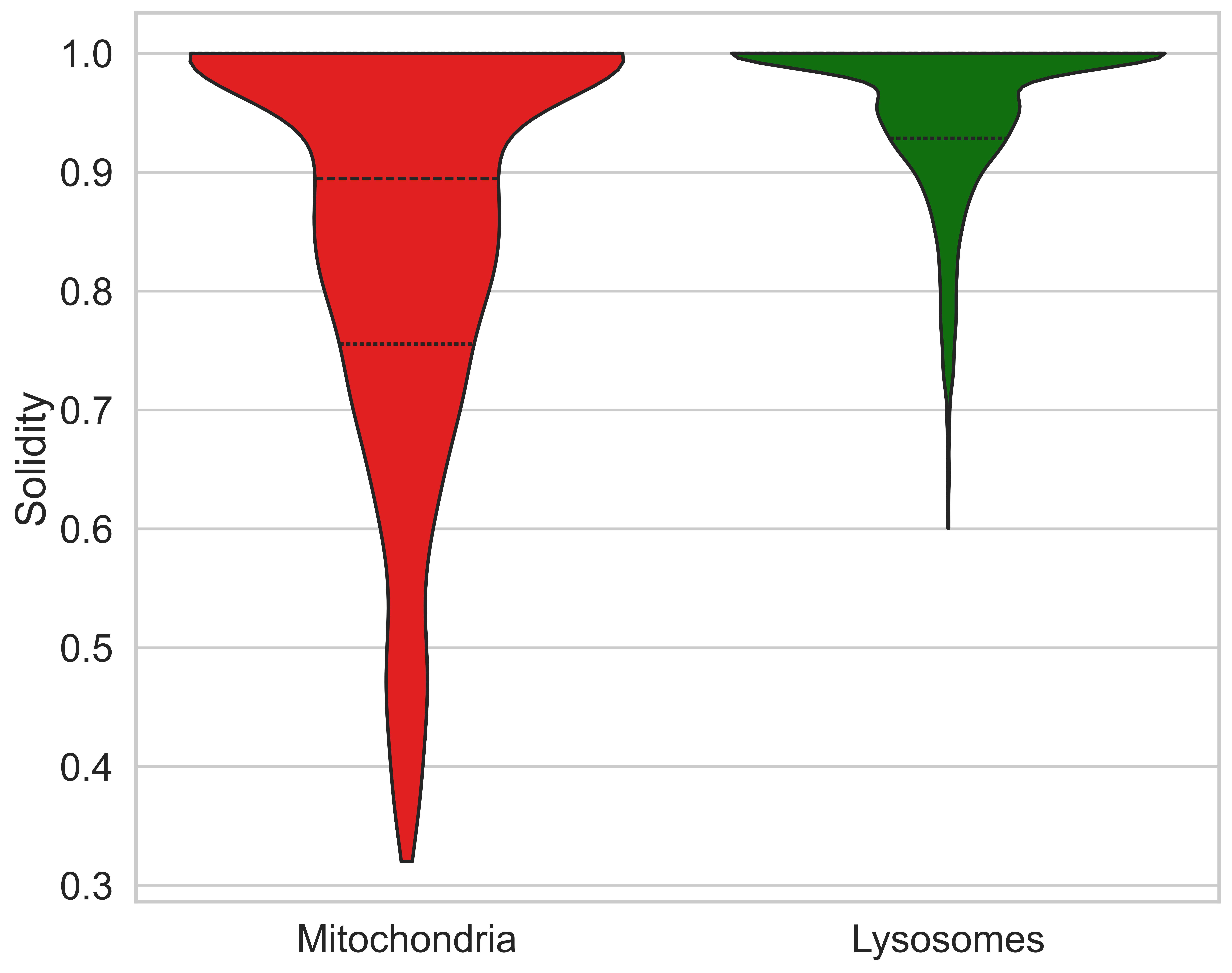

Supplement: Supplement 1 [file media-1.zip › Composite8/Step3.5_Shape_Profiling_Outputs/Step3_5_Solidity_ViolinPlot.png]

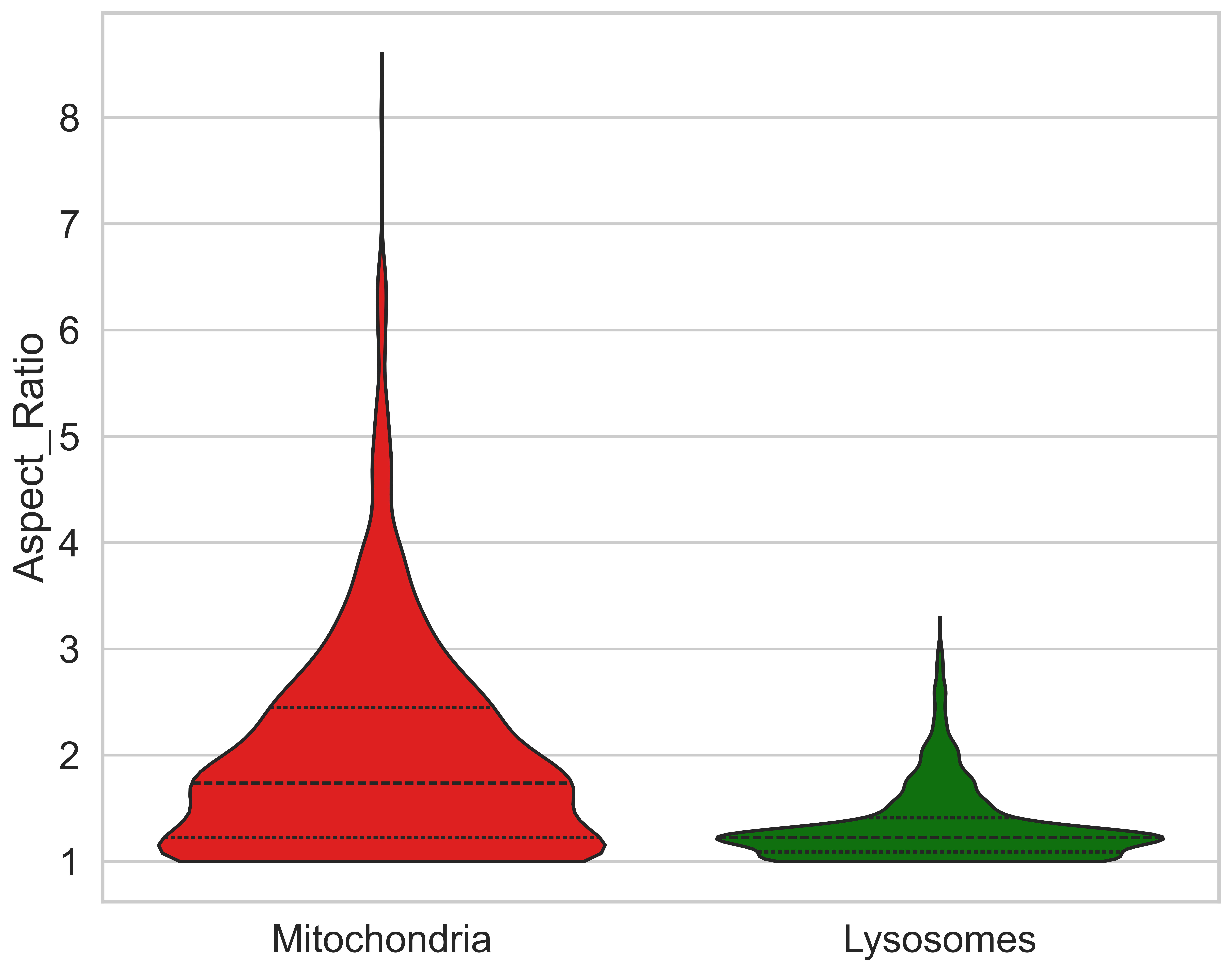

Supplement: Supplement 1 [file media-1.zip › Composite8/Step3.5_Shape_Profiling_Outputs/Step3_5_Aspect_Ratio_ViolinPlot.png]

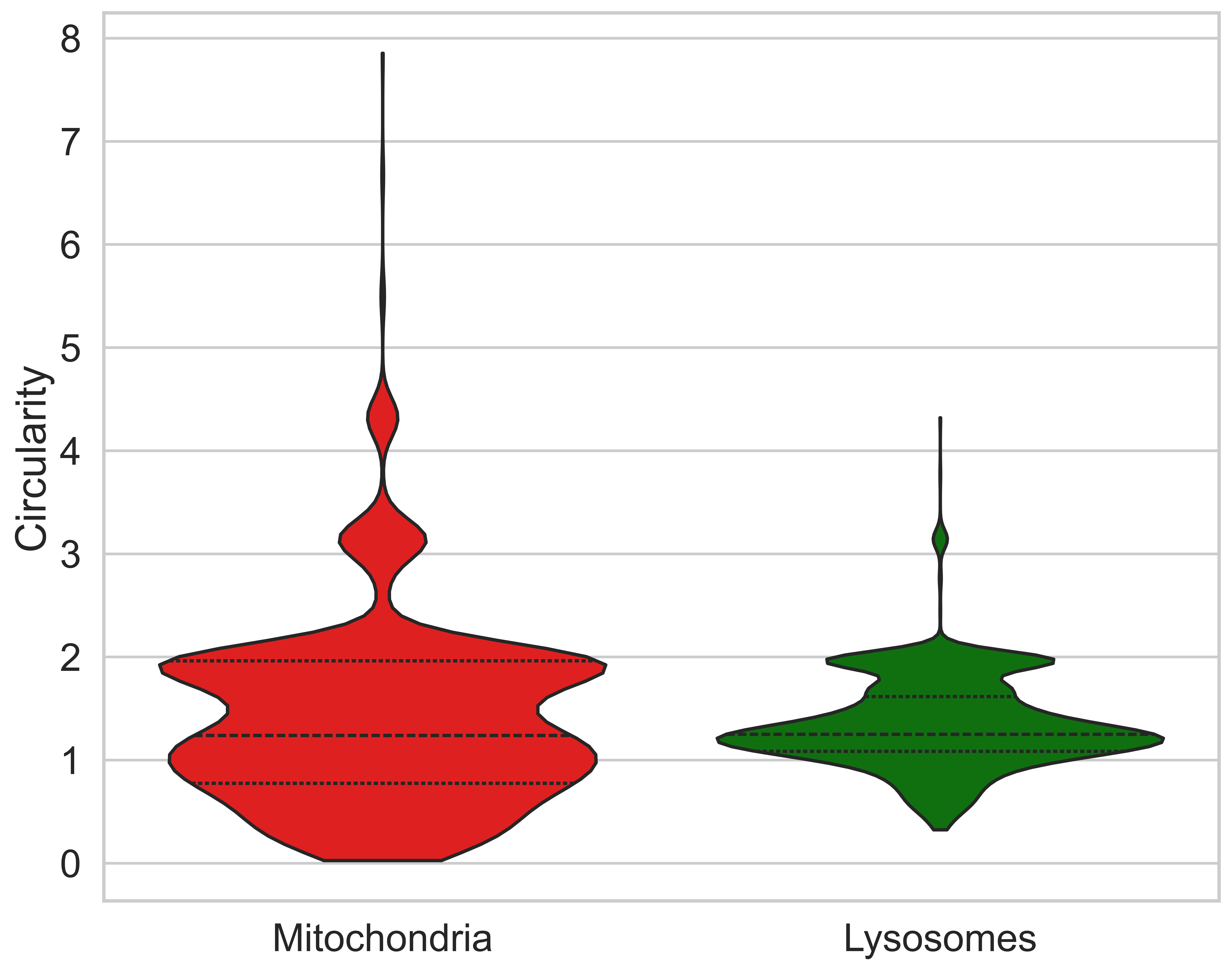

Supplement: Supplement 1 [file media-1.zip › Composite8/Step3.5_Shape_Profiling_Outputs/Step3_5_Circularity_ViolinPlot.png]

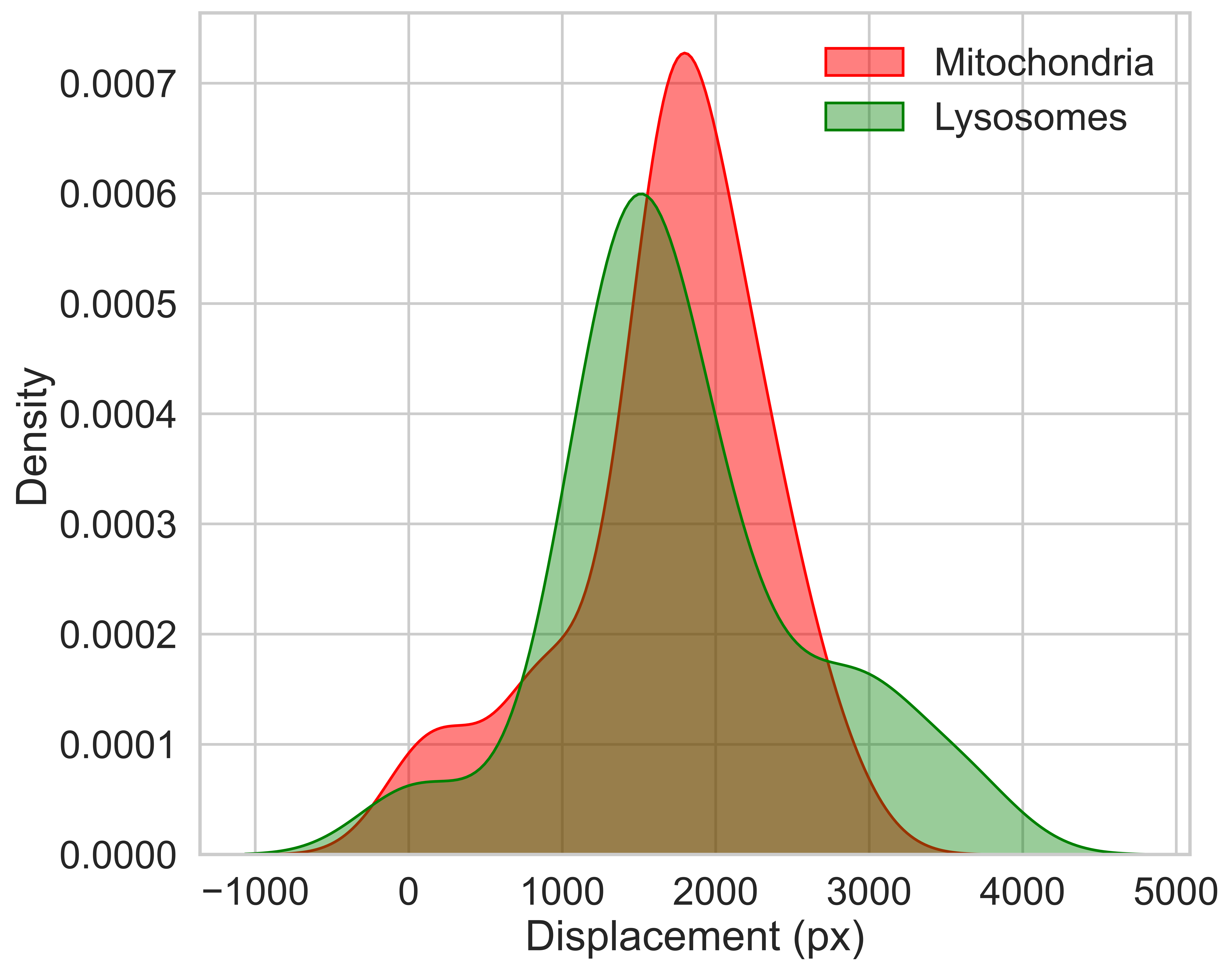

Supplement: Supplement 1 [file media-1.zip › Composite8/Step5_Motility_Outputs/Step5_Displacement_Distribution.png]

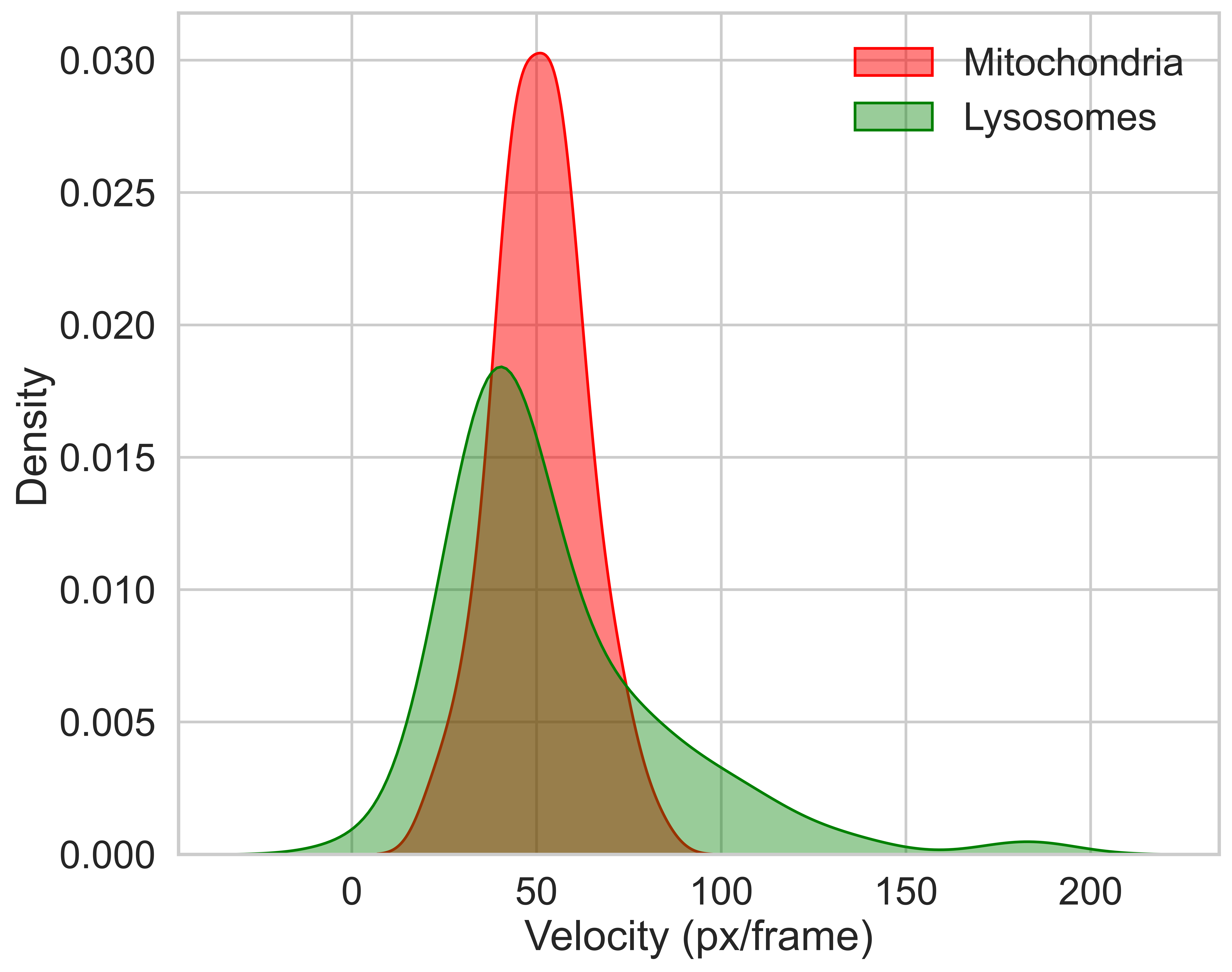

Supplement: Supplement 1 [file media-1.zip › Composite8/Step5_Motility_Outputs/Step5_Velocity_Distribution.png]

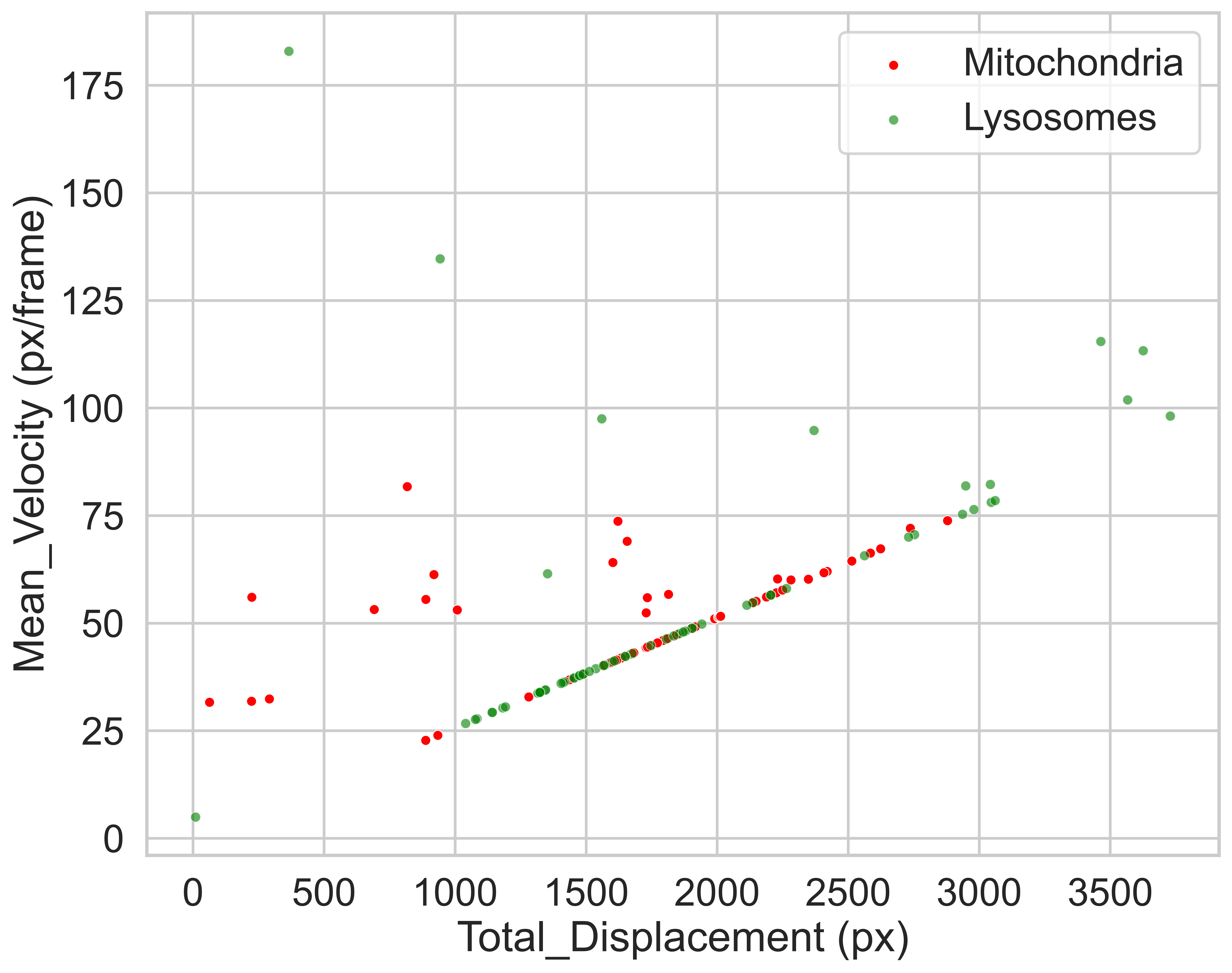

Supplement: Supplement 1 [file media-1.zip › Composite8/Step5_Motility_Outputs/Step5_Motility_Scatter.png]

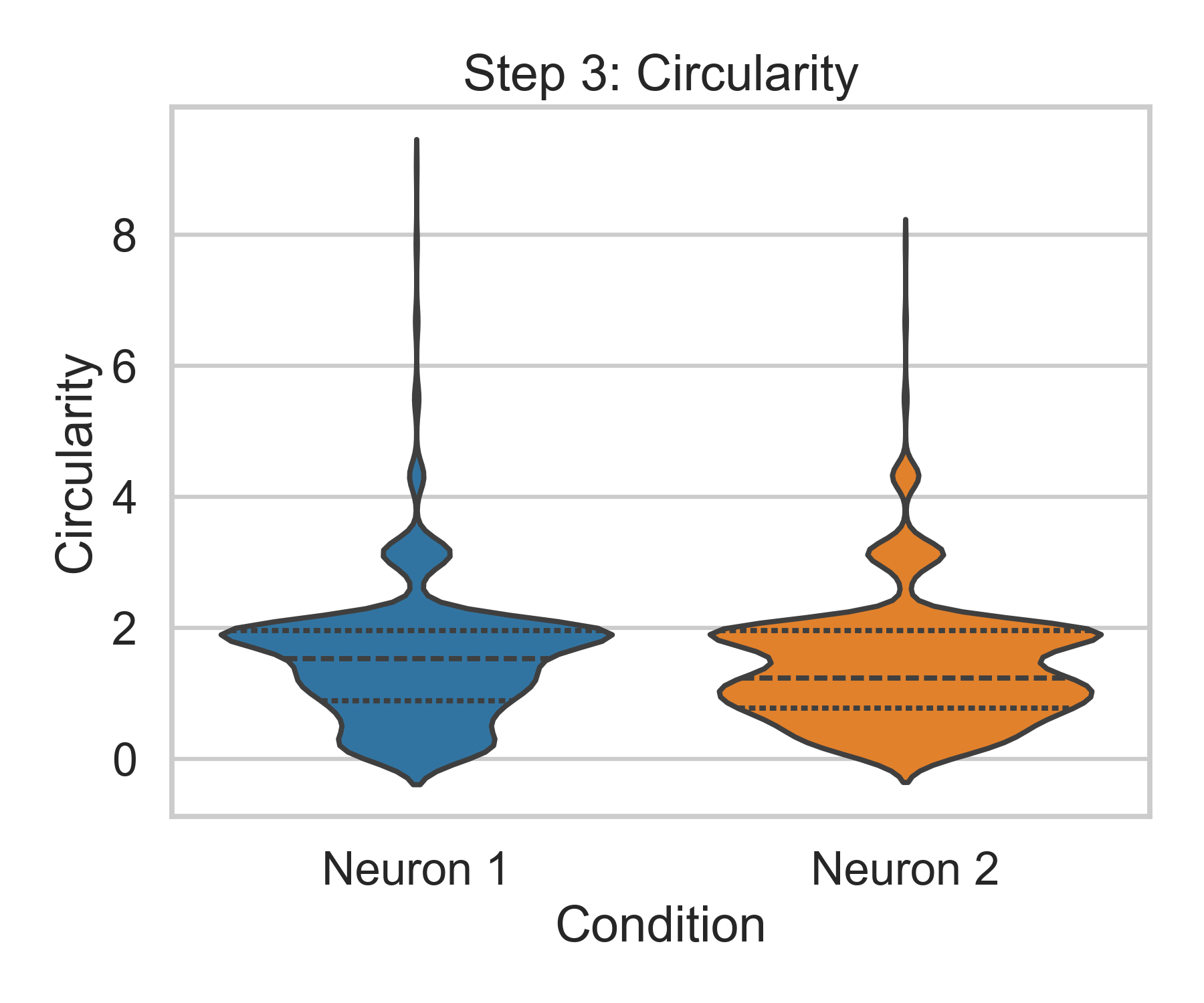

Supplement: Supplement 2 [file media-2.zip › AMTComparison Outputs/Step3_Circularity_Comparison.png]

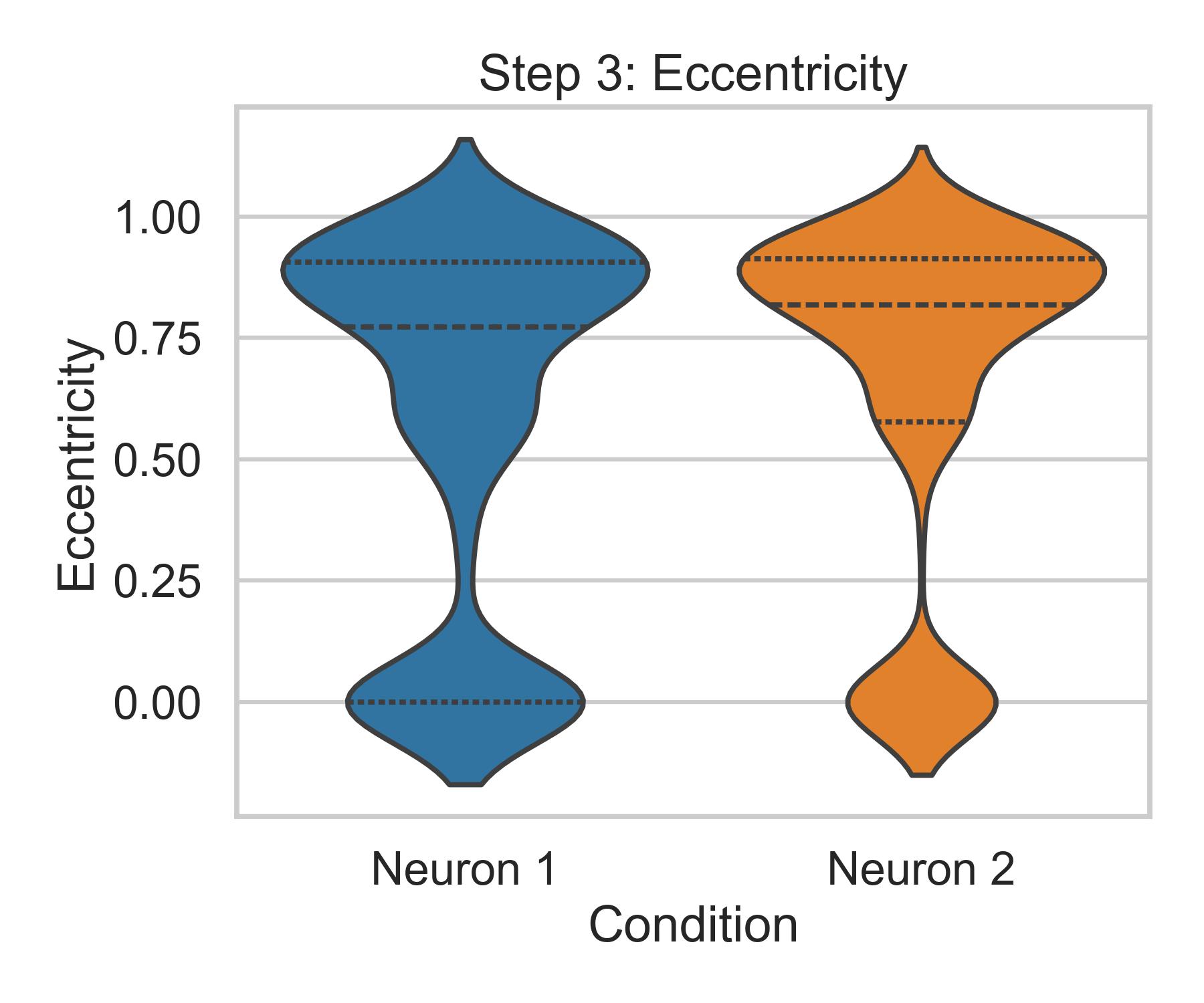

Supplement: Supplement 2 [file media-2.zip › AMTComparison Outputs/Step3_Eccentricity_Comparison.png]

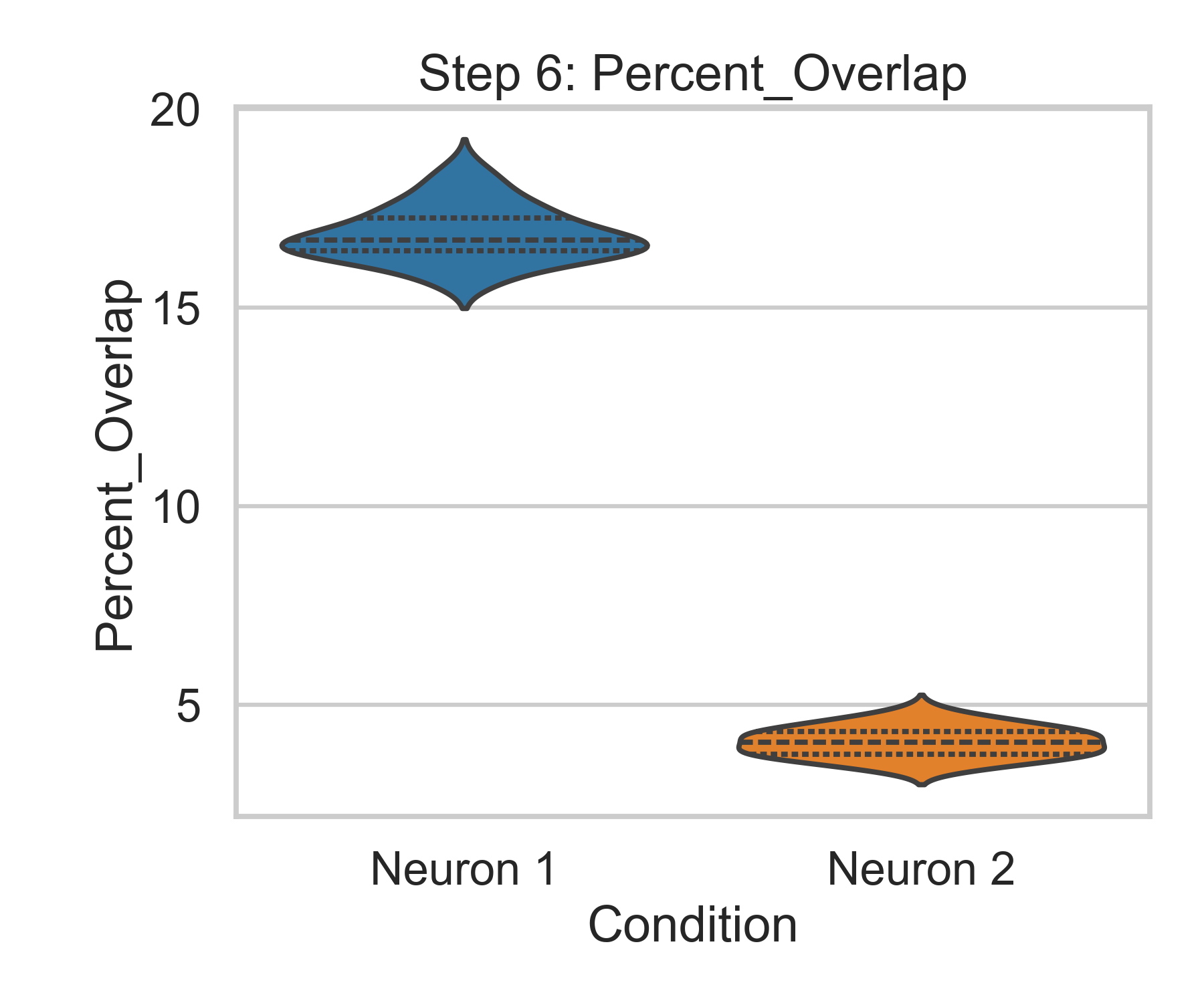

Supplement: Supplement 2 [file media-2.zip › AMTComparison Outputs/Step6_Percent_Overlap_Comparison.png]

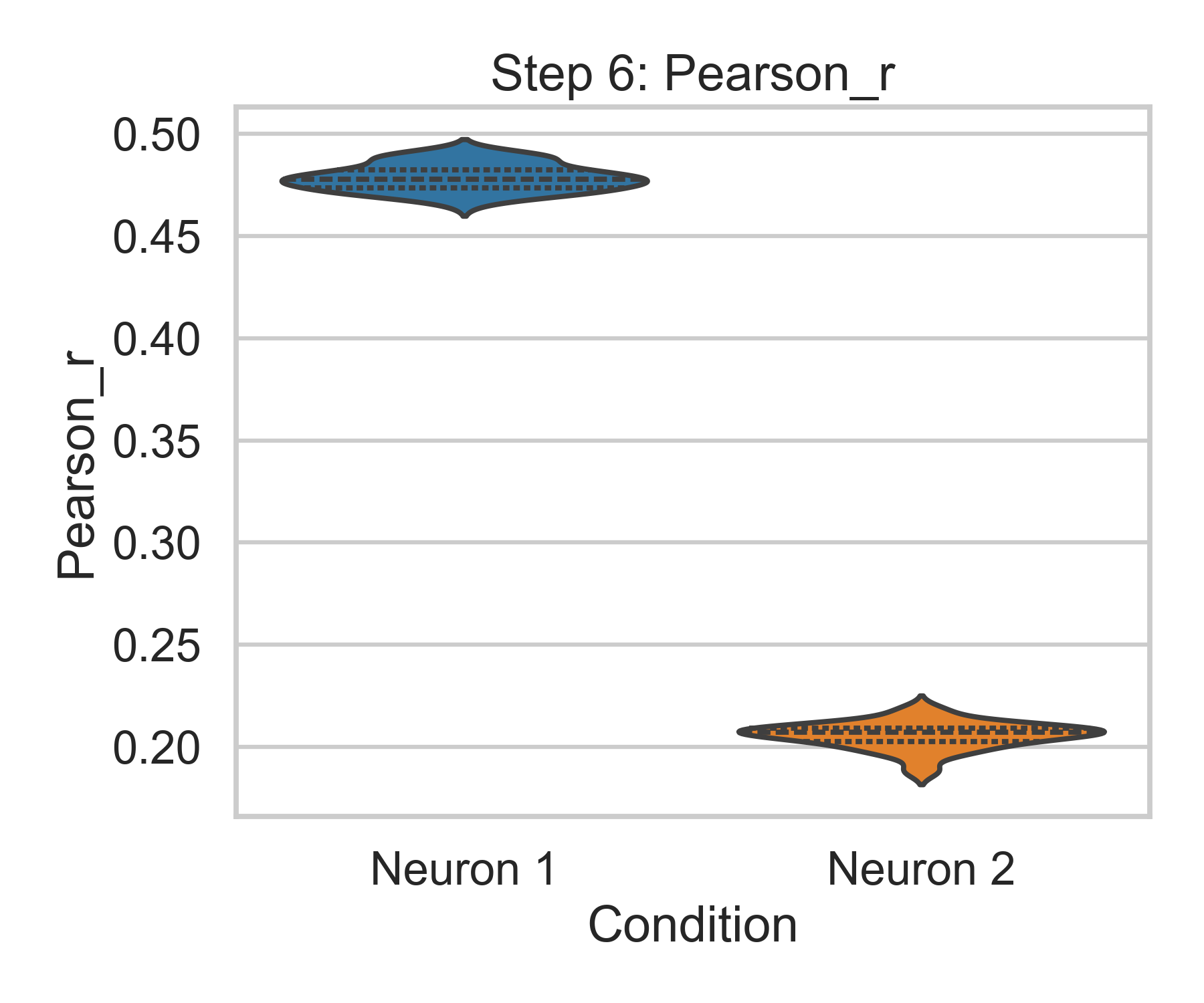

Supplement: Supplement 2 [file media-2.zip › AMTComparison Outputs/Step6_Pearson_r_Comparison.png]

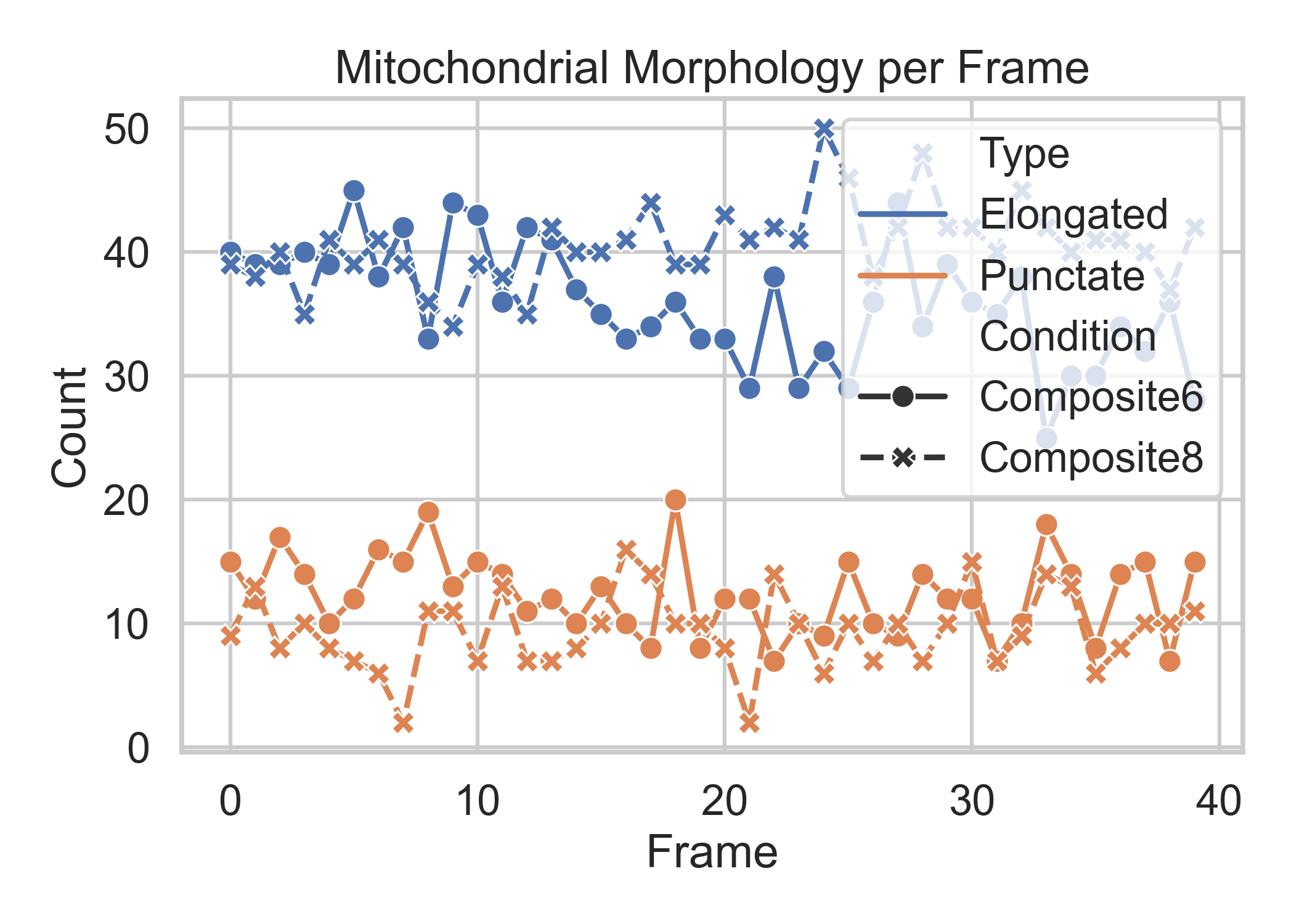

Supplement: Supplement 2 [file media-2.zip › AMTComparison Outputs/Step2_Morphology_Comparison.png]

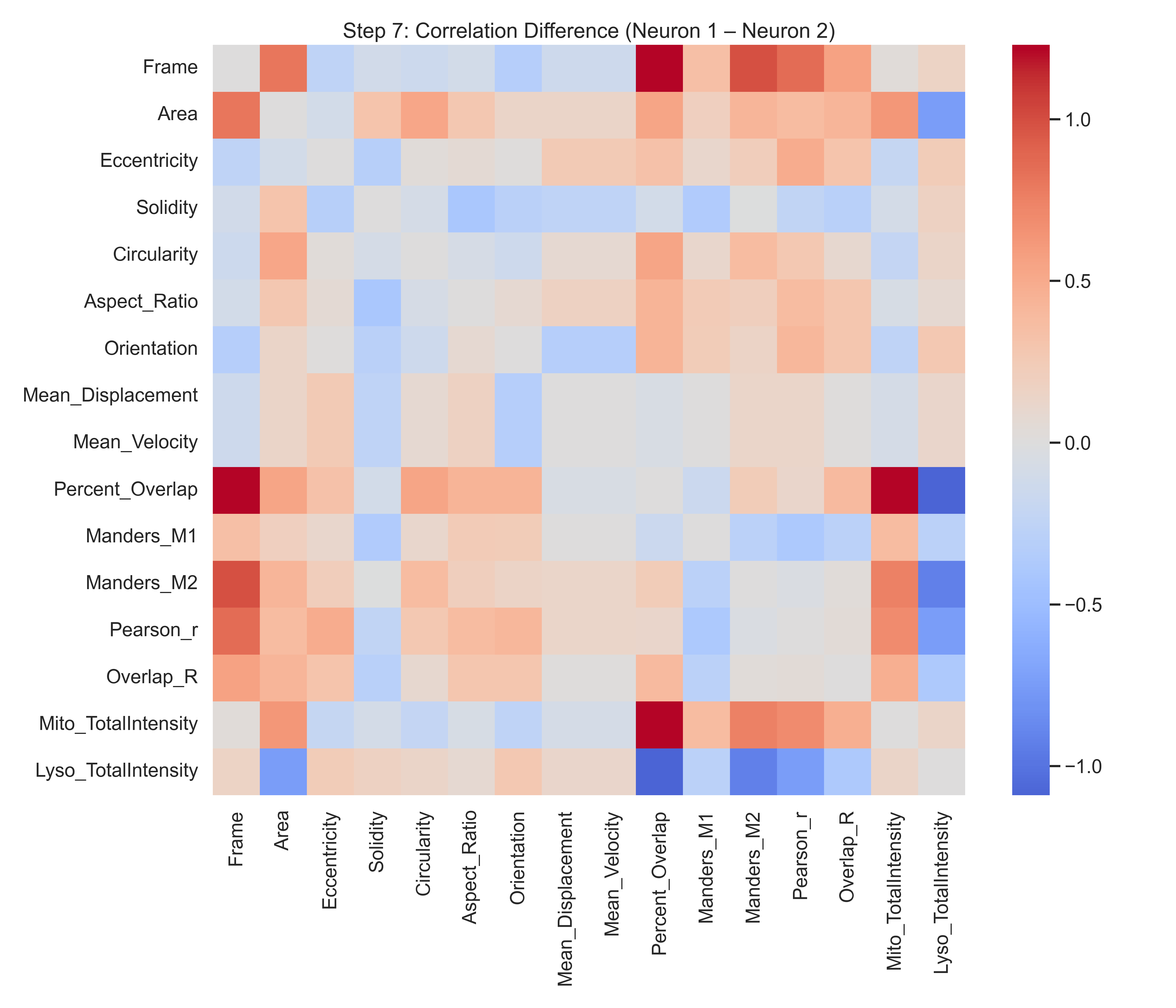

Supplement: Supplement 2 [file media-2.zip › AMTComparison Outputs/Step7_CorrelationMatrix_Difference_Neuron1_vs_Neuron2.png]

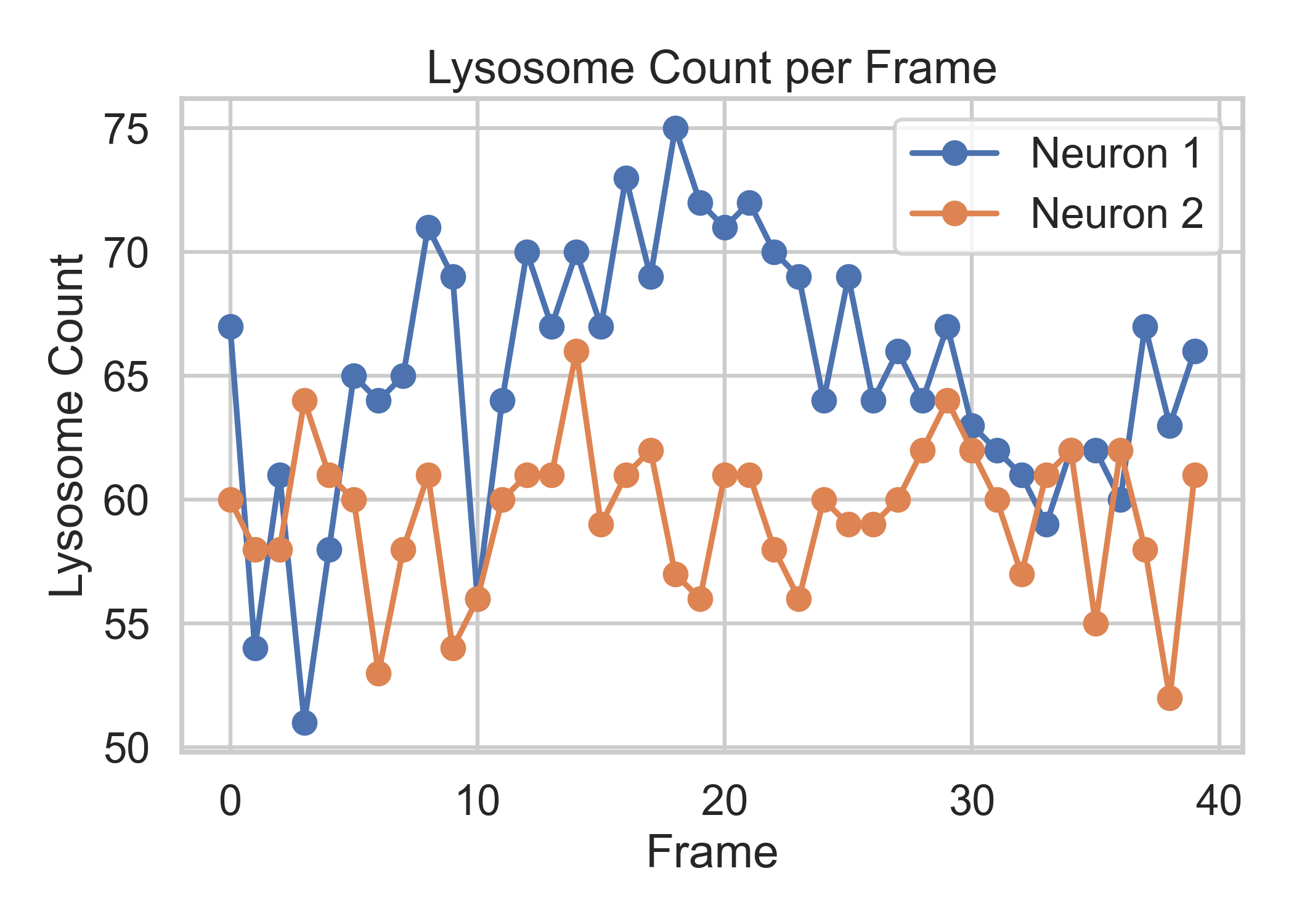

Supplement: Supplement 2 [file media-2.zip › AMTComparison Outputs/Step1_LysoCount_Comparison.png]

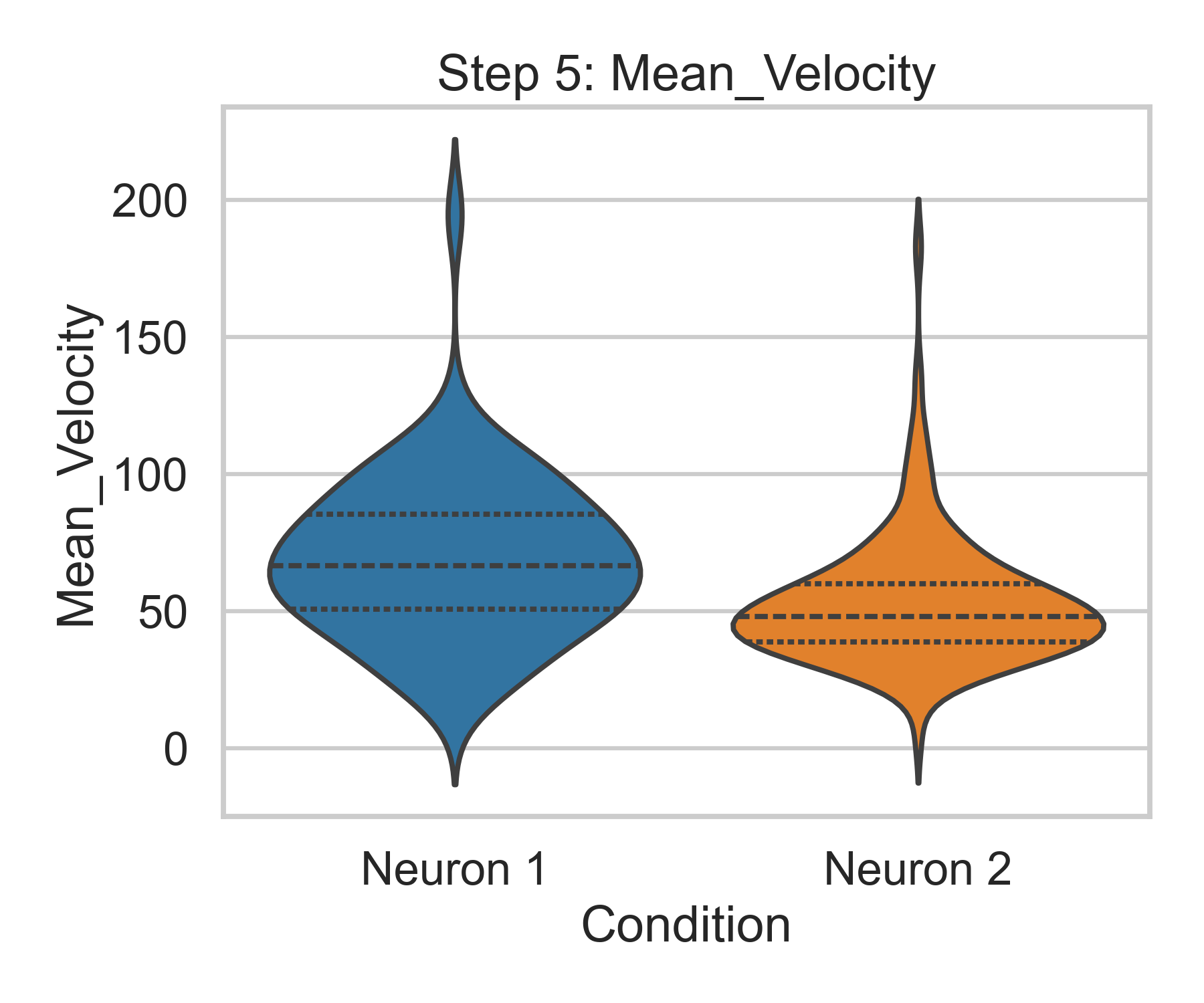

Supplement: Supplement 2 [file media-2.zip › AMTComparison Outputs/Step5_Mean_Velocity_Comparison.png]

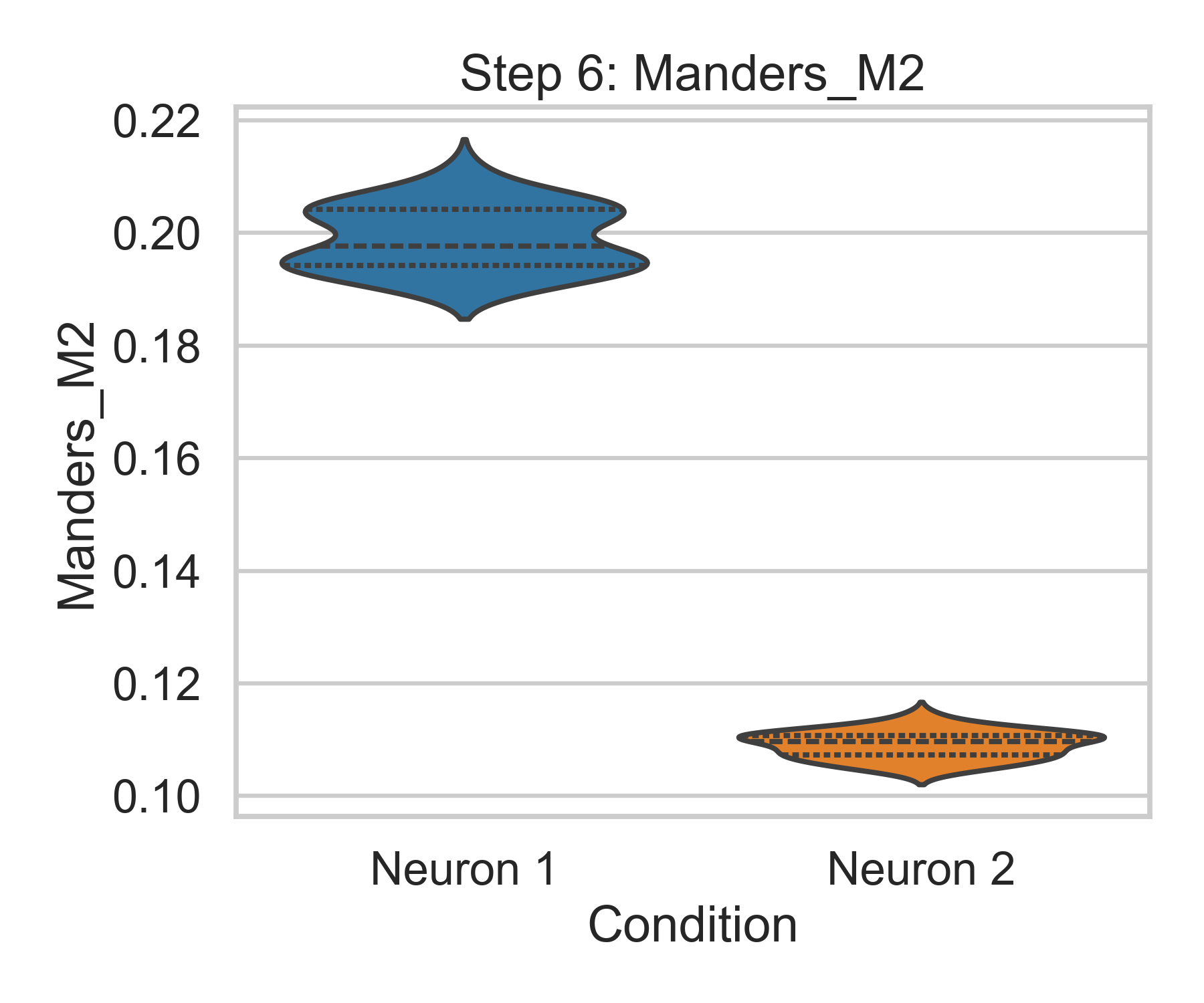

Supplement: Supplement 2 [file media-2.zip › AMTComparison Outputs/Step6_Manders_M2_Comparison.png]

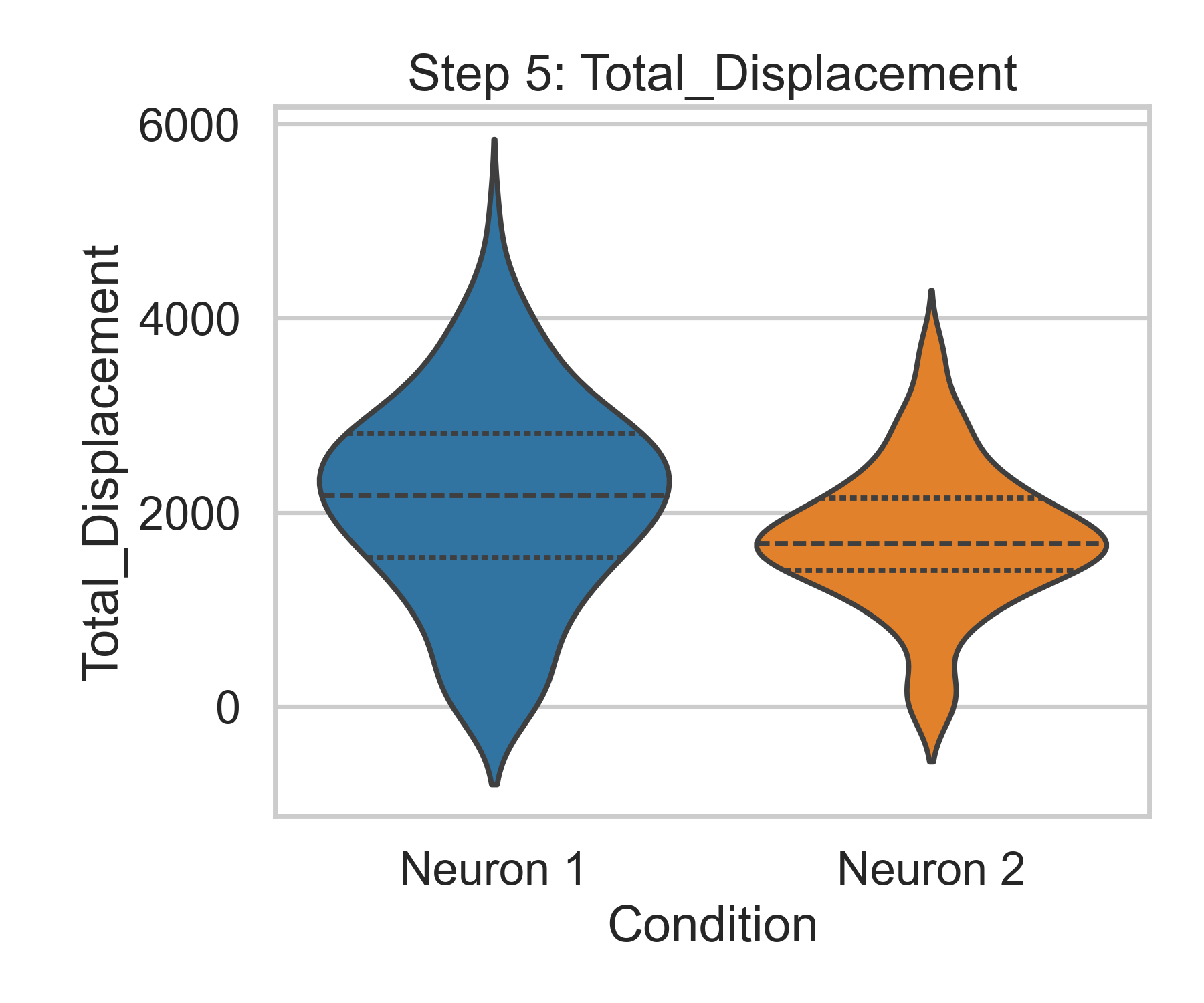

Supplement: Supplement 2 [file media-2.zip › AMTComparison Outputs/Step5_Total_Displacement_Comparison.png]

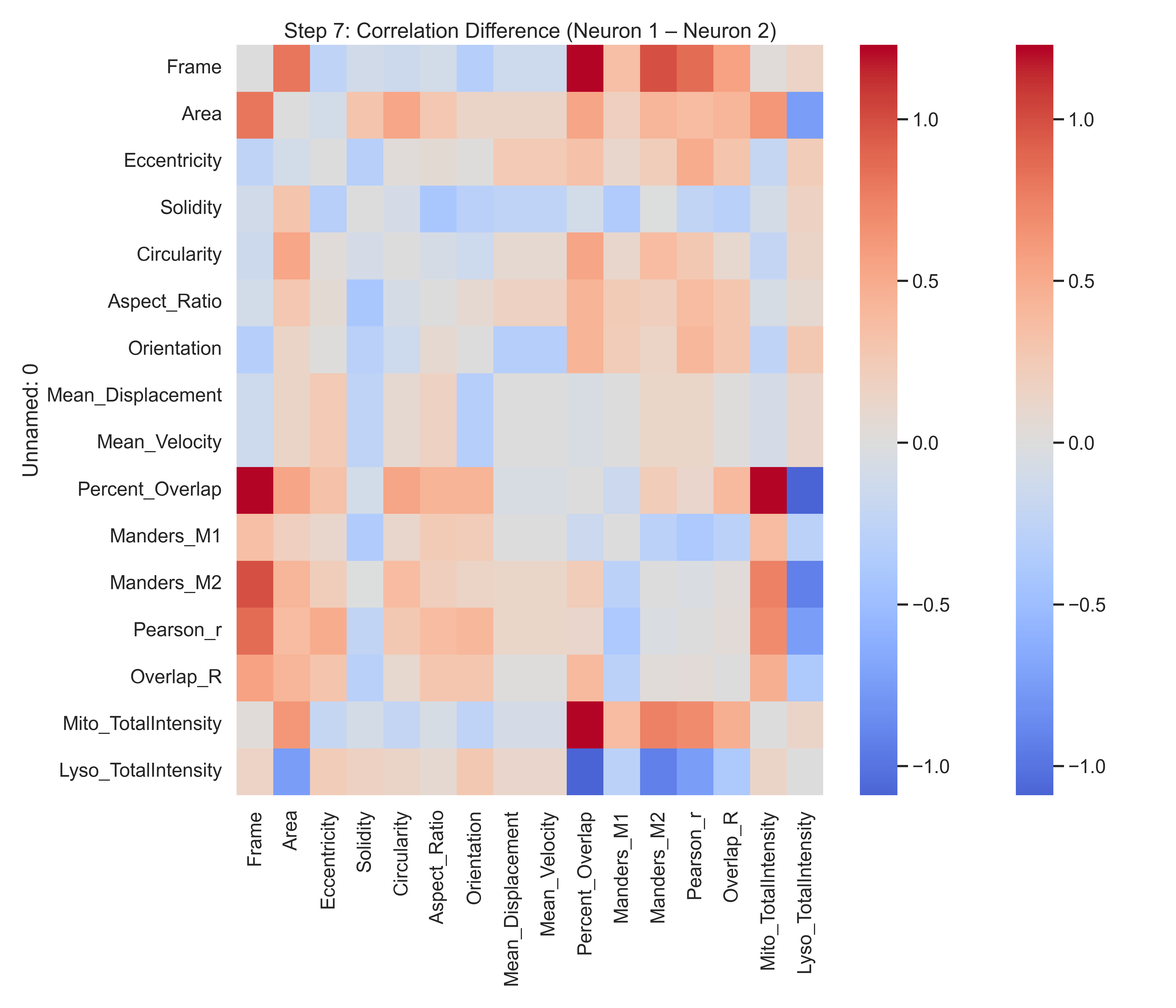

Supplement: Supplement 2 [file media-2.zip › AMTComparison Outputs/Step7_CorrelationMatrix_Difference.png]

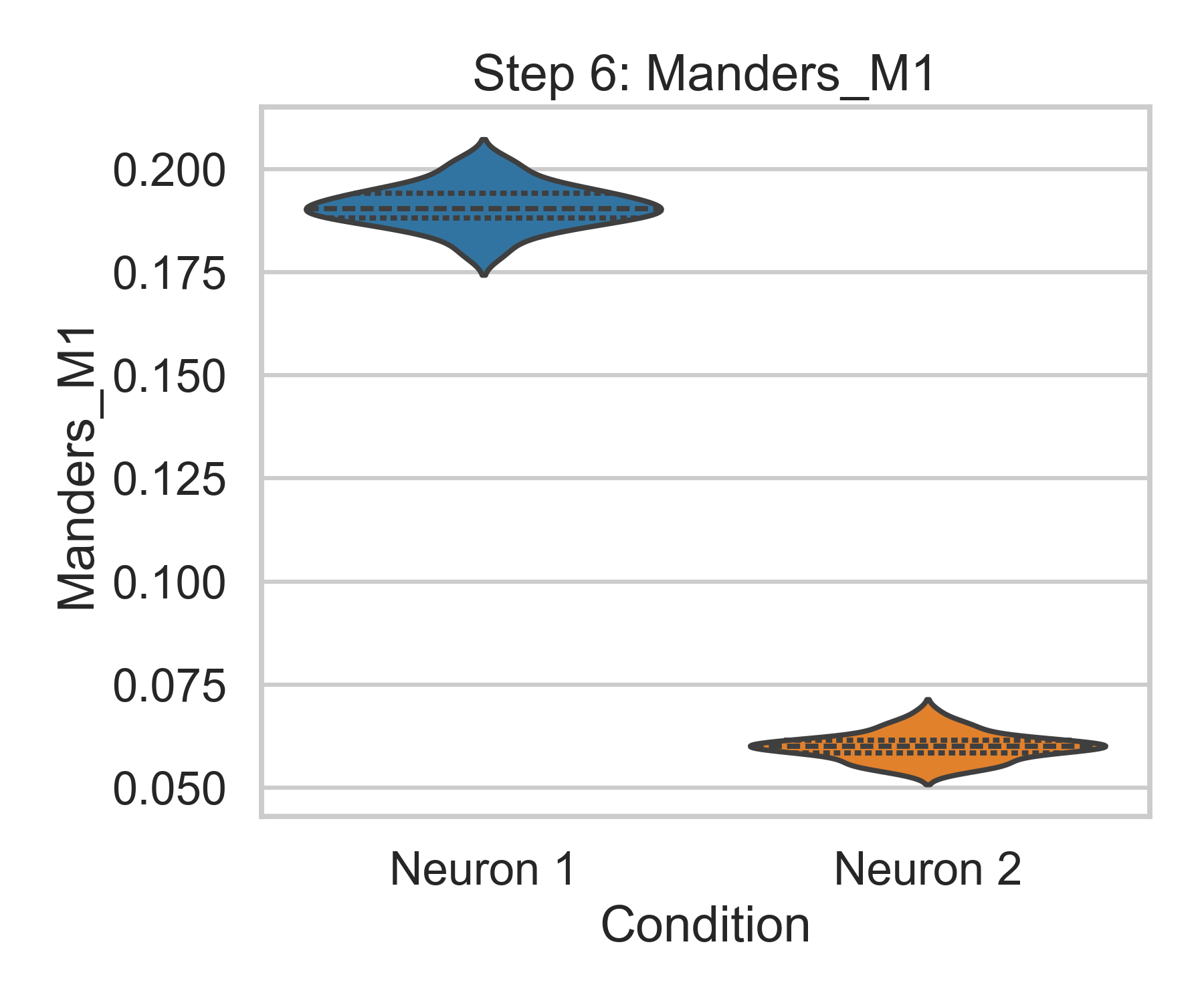

Supplement: Supplement 2 [file media-2.zip › AMTComparison Outputs/Step6_Manders_M1_Comparison.png]

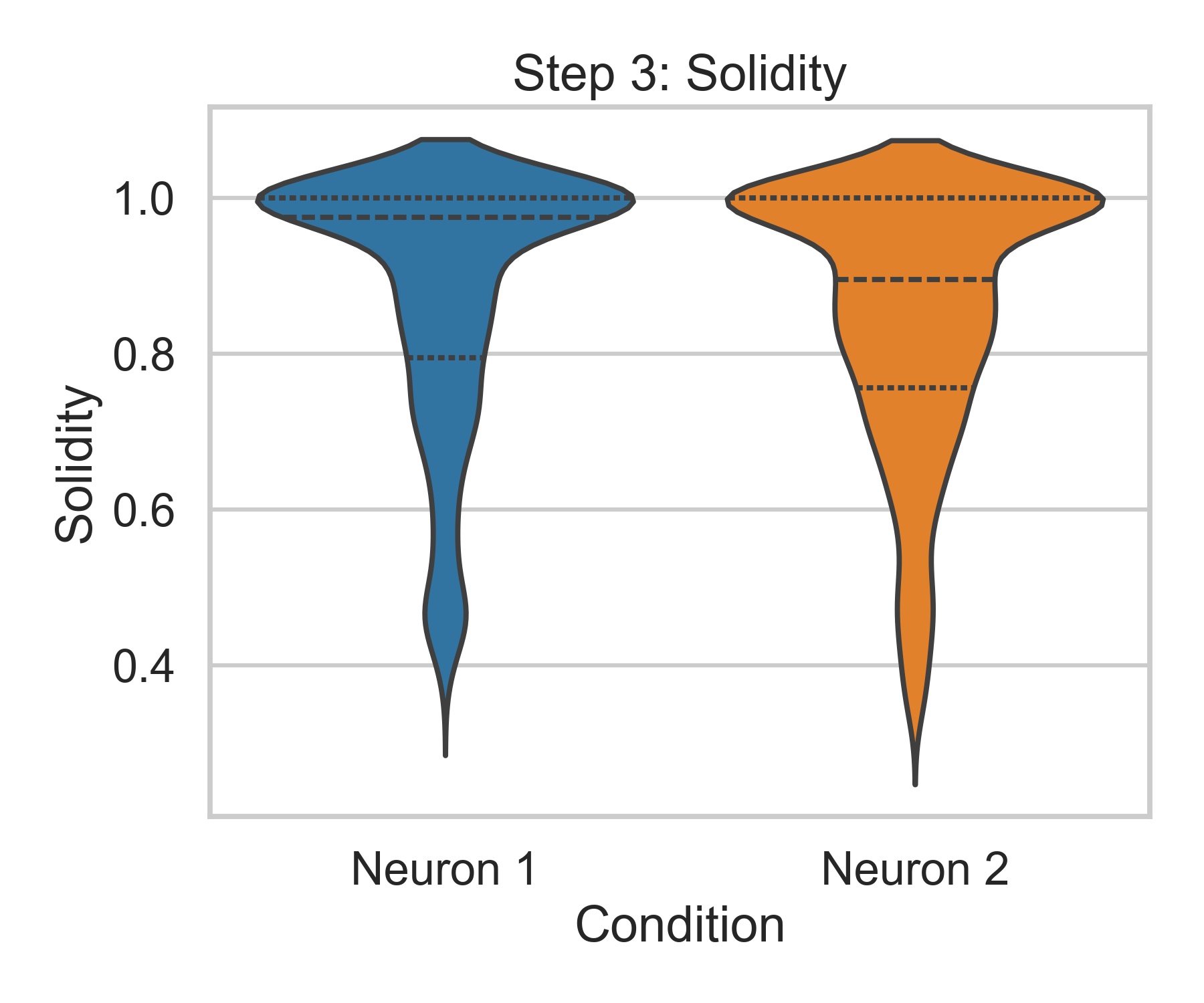

Supplement: Supplement 2 [file media-2.zip › AMTComparison Outputs/Step3_Solidity_Comparison.png]

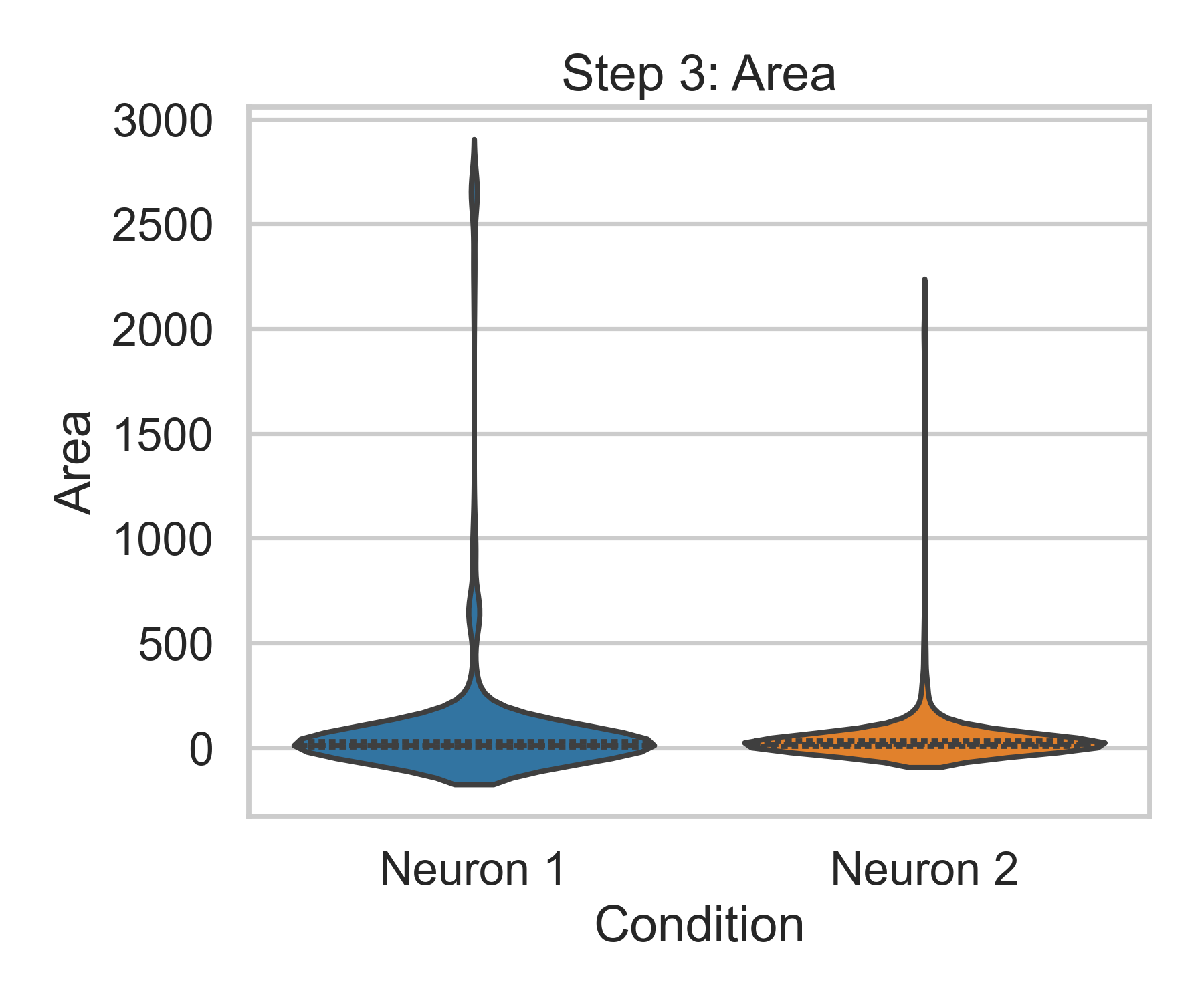

Supplement: Supplement 2 [file media-2.zip › AMTComparison Outputs/Step3_Area_Comparison.png]

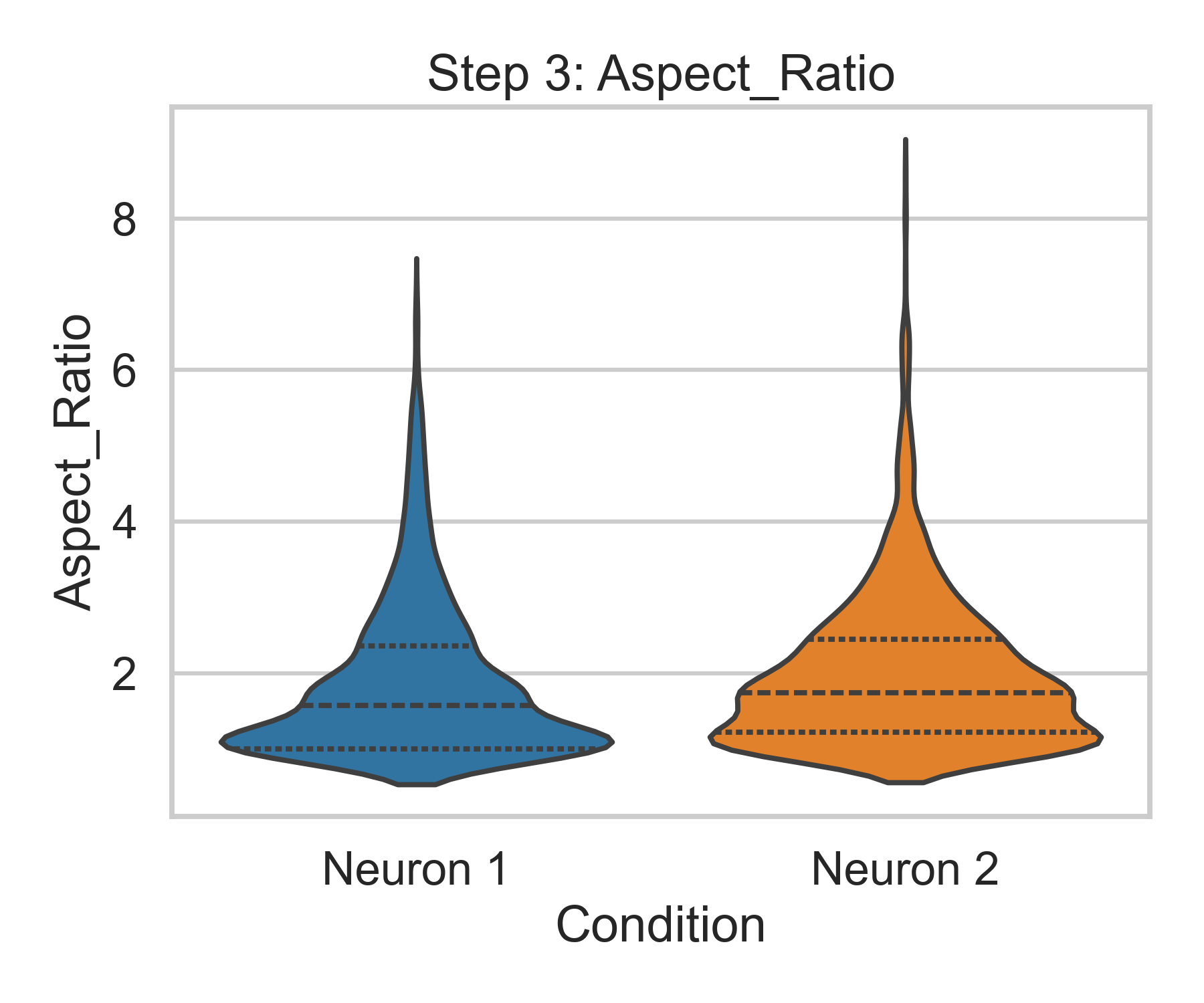

Supplement: Supplement 2 [file media-2.zip › AMTComparison Outputs/Step3_Aspect_Ratio_Comparison.png]
