# Supplementary material for: AutoMorphoTrack: A modular framework for quantitative analysis of organelle morphology, motility, and interactions at single-cell resolution
Source: bioRxiv. 2026 Feb 2:2025.07.19.665650. Preprint. [Version 5] doi: 10.1101/2025.07.19.665650 (PMC12889450; doi:10.1101/2025.07.19.665650)
Supplement: Supplement 4 [file NIHPP2025.07.19.665650v5-supplement-4.pdf]

bioRxiv preprint doi: https://doi.org/10.1101/2025.07.19.665650; this version posted February 2, 2026. The copyright holder for this preprint (which was not certified by peer review) is the author/funder, who has granted bioRxiv a license to display the preprint in perpetuity. It is made available under aCC-BY 4.0 International license.

Supplementary Figures

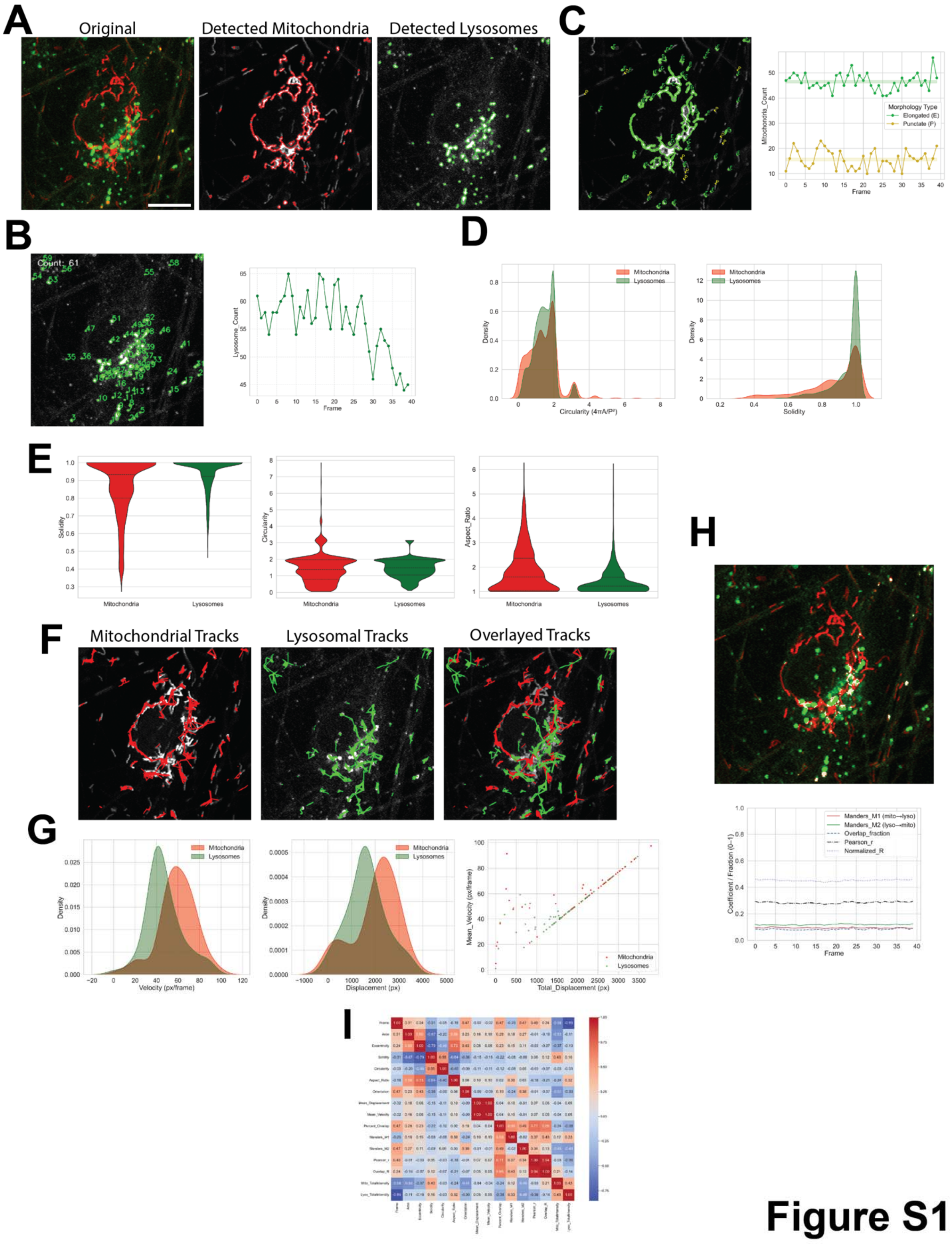

Figure S1

bioRxiv preprint doi: https://doi.org/10.1101/2025.07.19.665650; this version posted February 2, 2026. The copyright holder for this preprint (which was not certified by peer review) is the author/funder, who has granted bioRxiv a license to display the preprint in perpetuity. It is made available under aCC-BY 4.0 International license.

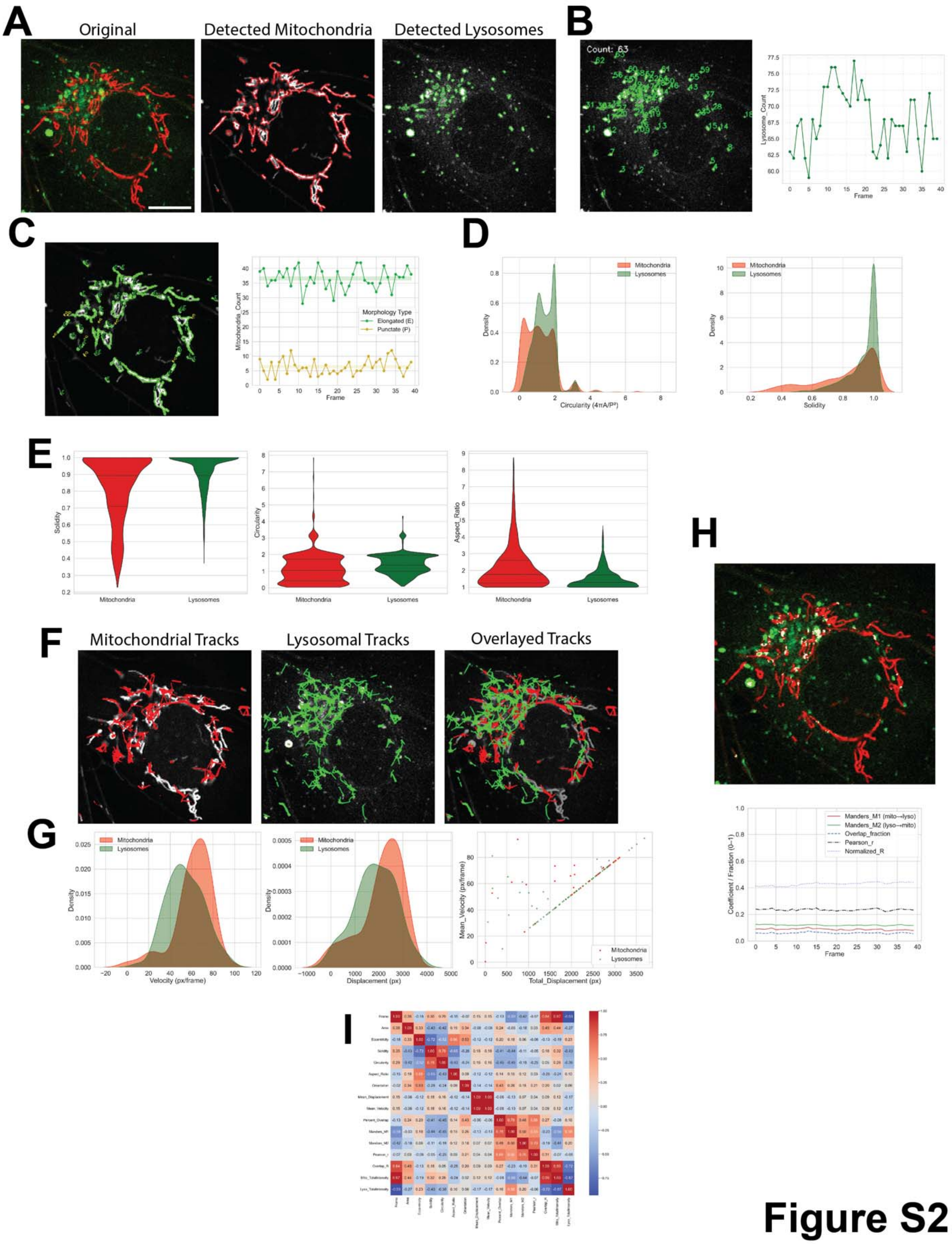

Figure S2

bioRxiv preprint doi: https://doi.org/10.1101/2025.07.19.665650; this version posted February 2, 2026. The copyright holder for this preprint (which was not certified by peer review) is the author/funder, who has granted bioRxiv a license to display the preprint in perpetuity. It is made available under aCC-BY 4.0 International license.

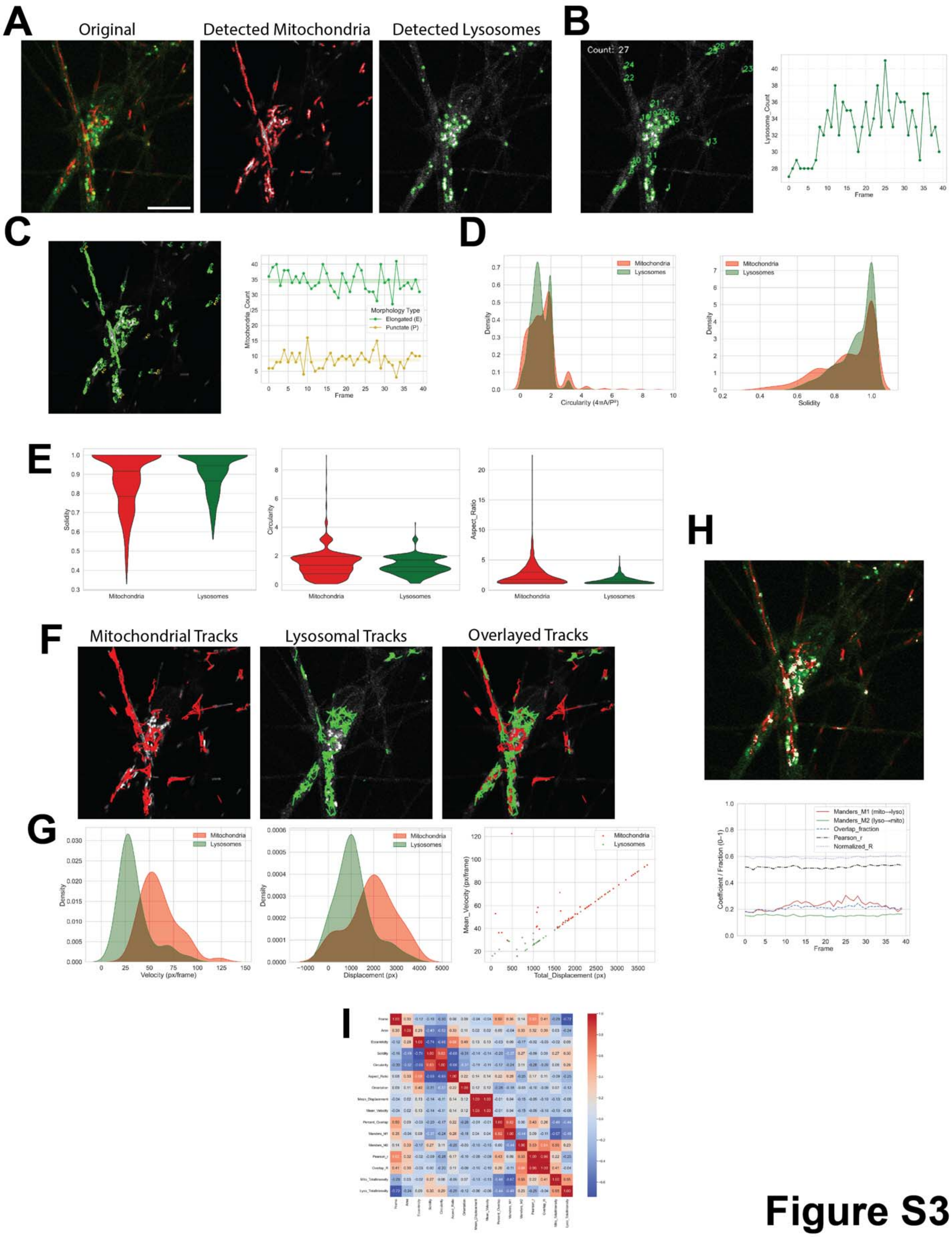

Figure S3

bioRxiv preprint doi: https://doi.org/10.1101/2025.07.19.665650; this version posted February 2, 2026. The copyright holder for this preprint (which was not certified by peer review) is the author/funder, who has granted bioRxiv a license to display the preprint in perpetuity. It is made available under aCC-BY 4.0 International license.

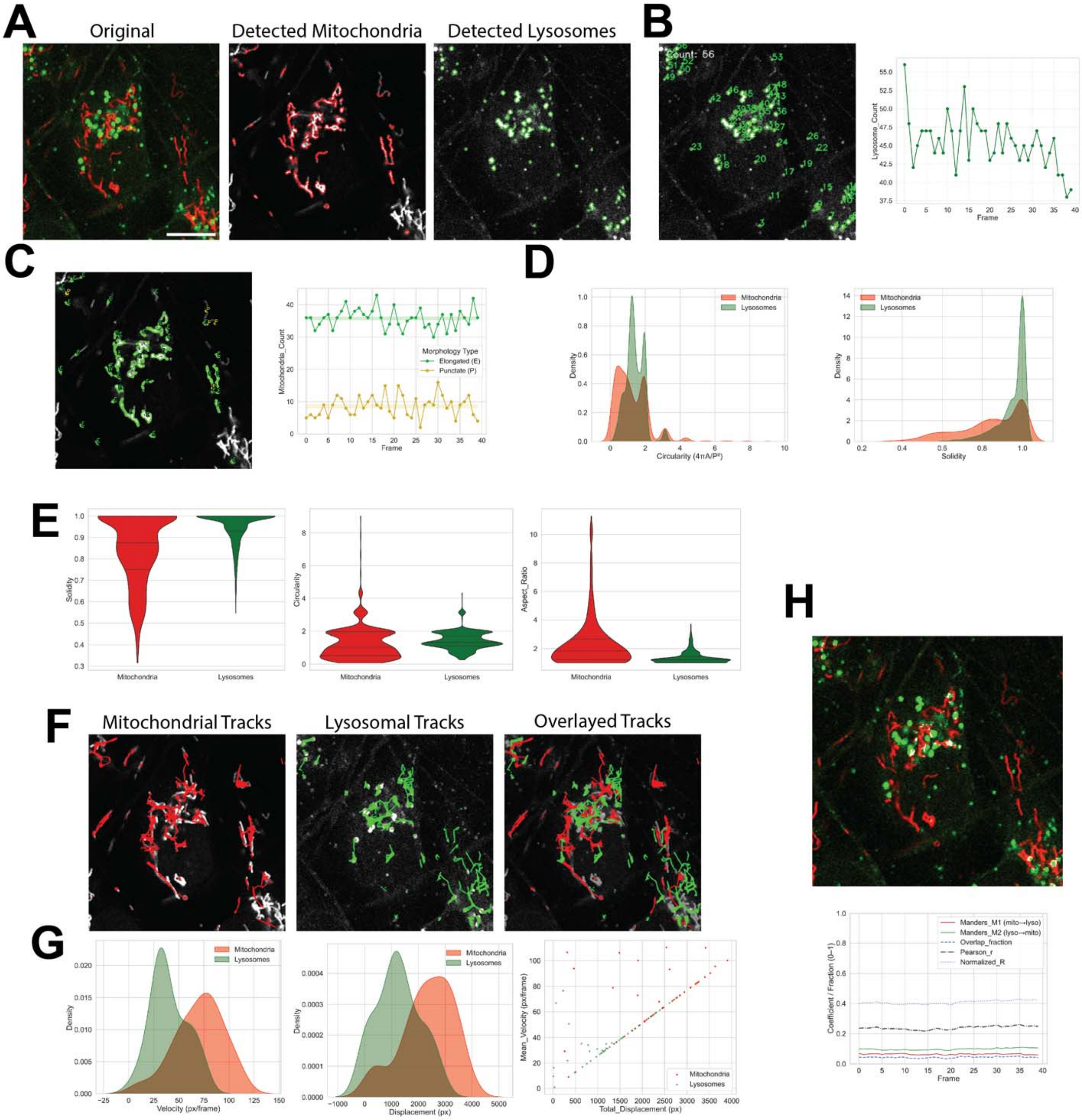

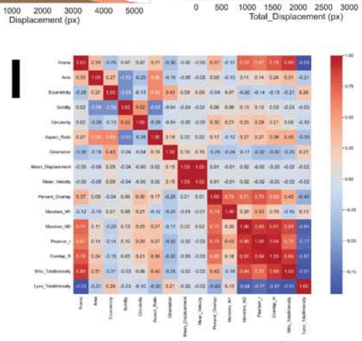

Figure S4

bioRxiv preprint doi: https://doi.org/10.1101/2025.07.19.665650; this version posted February 2, 2026. The copyright holder for this preprint (which was not certified by peer review) is the author/funder, who has granted bioRxiv a license to display the preprint in perpetuity. It is made available under aCC-BY 4.0 International license.

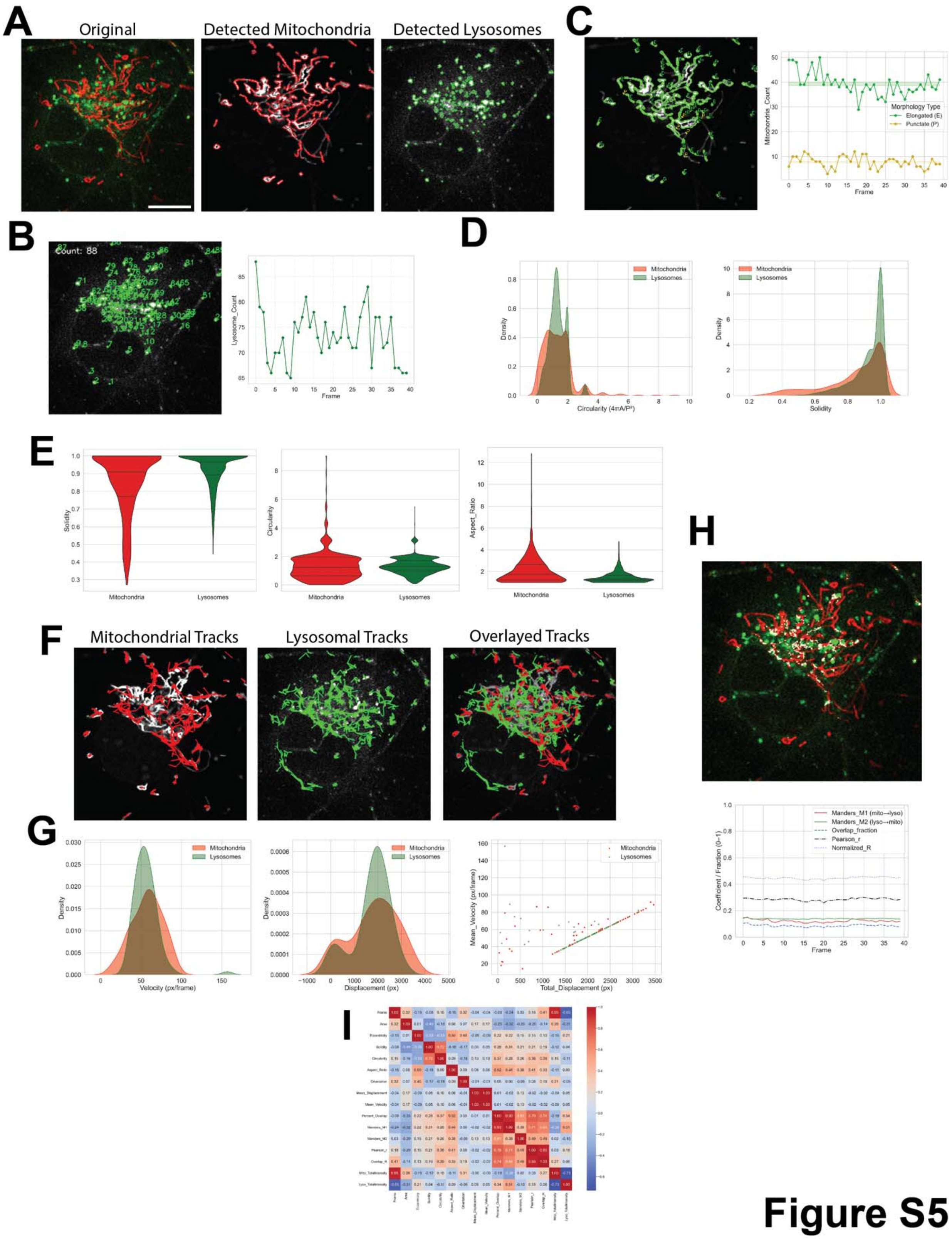

Figure S5

bioRxiv preprint doi: https://doi.org/10.1101/2025.07.19.665650; this version posted February 2, 2026. The copyright holder for this preprint (which was not certified by peer review) is the author/funder, who has granted bioRxiv a license to display the preprint in perpetuity. It is made available under aCC-BY 4.0 International license.

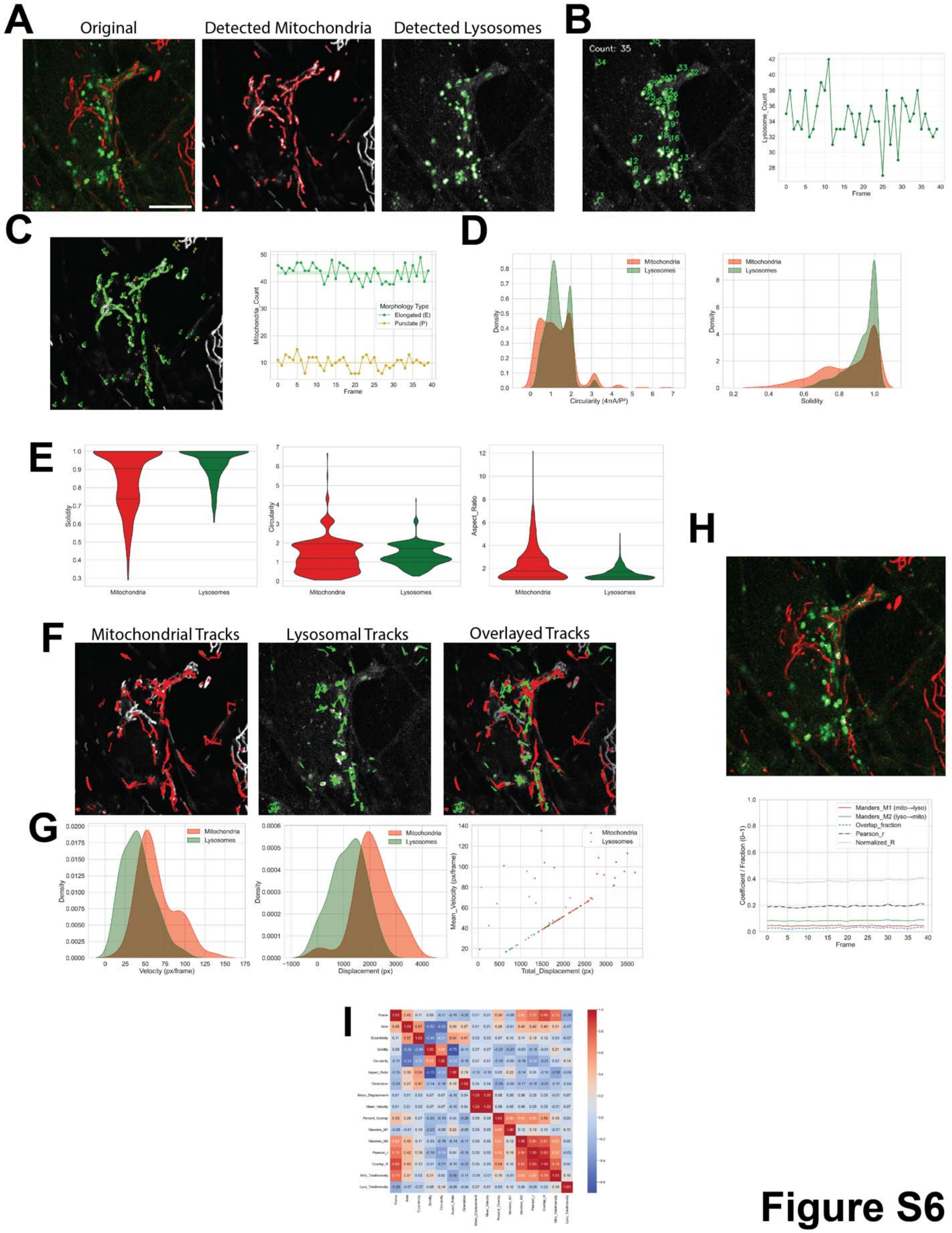

Figure S6

bioRxiv preprint doi: https://doi.org/10.1101/2025.07.19.665650; this version posted February 2, 2026. The copyright holder for this preprint (which was not certified by peer review) is the author/funder, who has granted bioRxiv a license to display the preprint in perpetuity. It is made available under aCC-BY 4.0 International license.

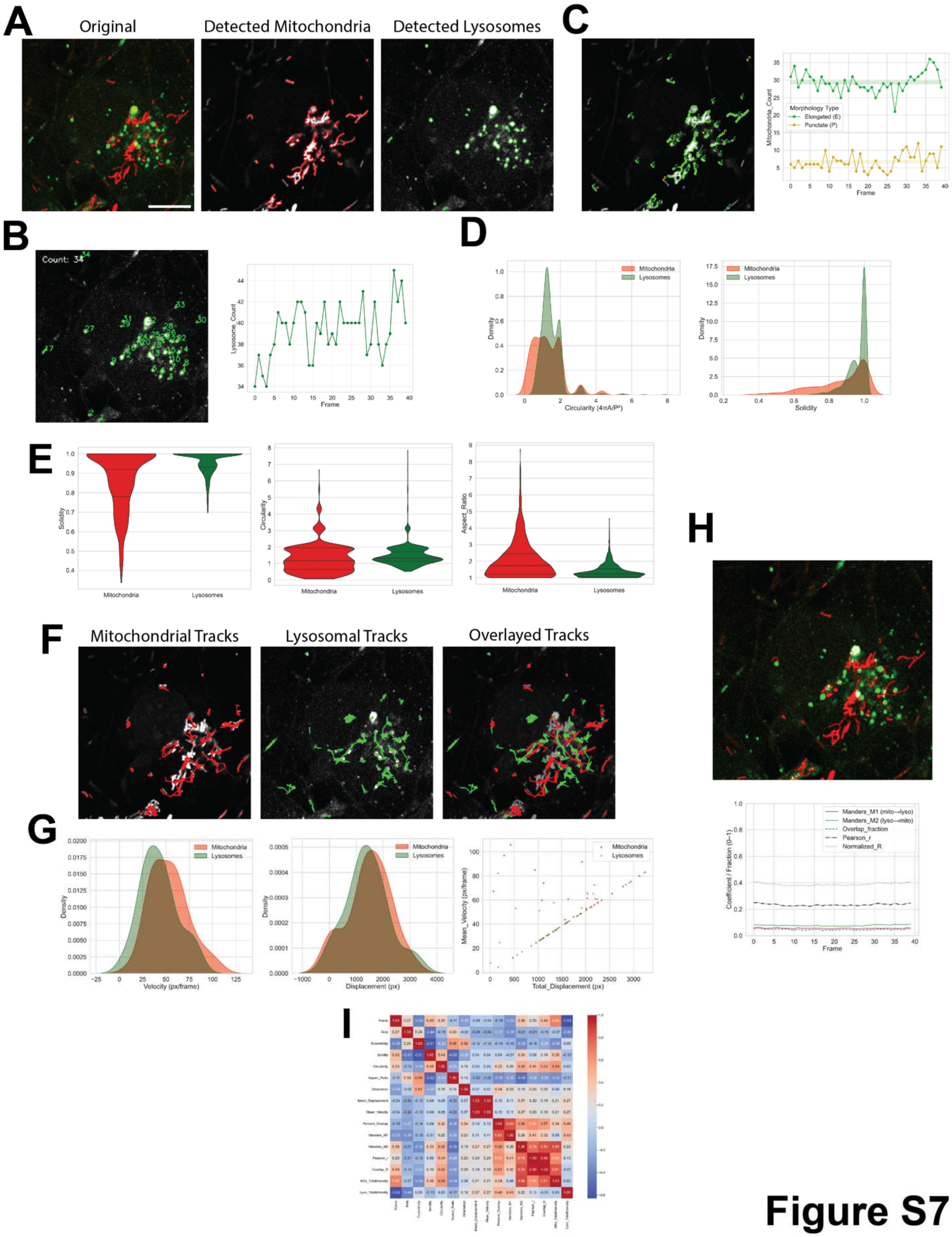

Figure S7

bioRxiv preprint doi: https://doi.org/10.1101/2025.07.19.665650; this version posted February 2, 2026. The copyright holder for this preprint (which was not certified by peer review) is the author/funder, who has granted bioRxiv a license to display the preprint in perpetuity. It is made available under aCC-BY 4.0 International license.

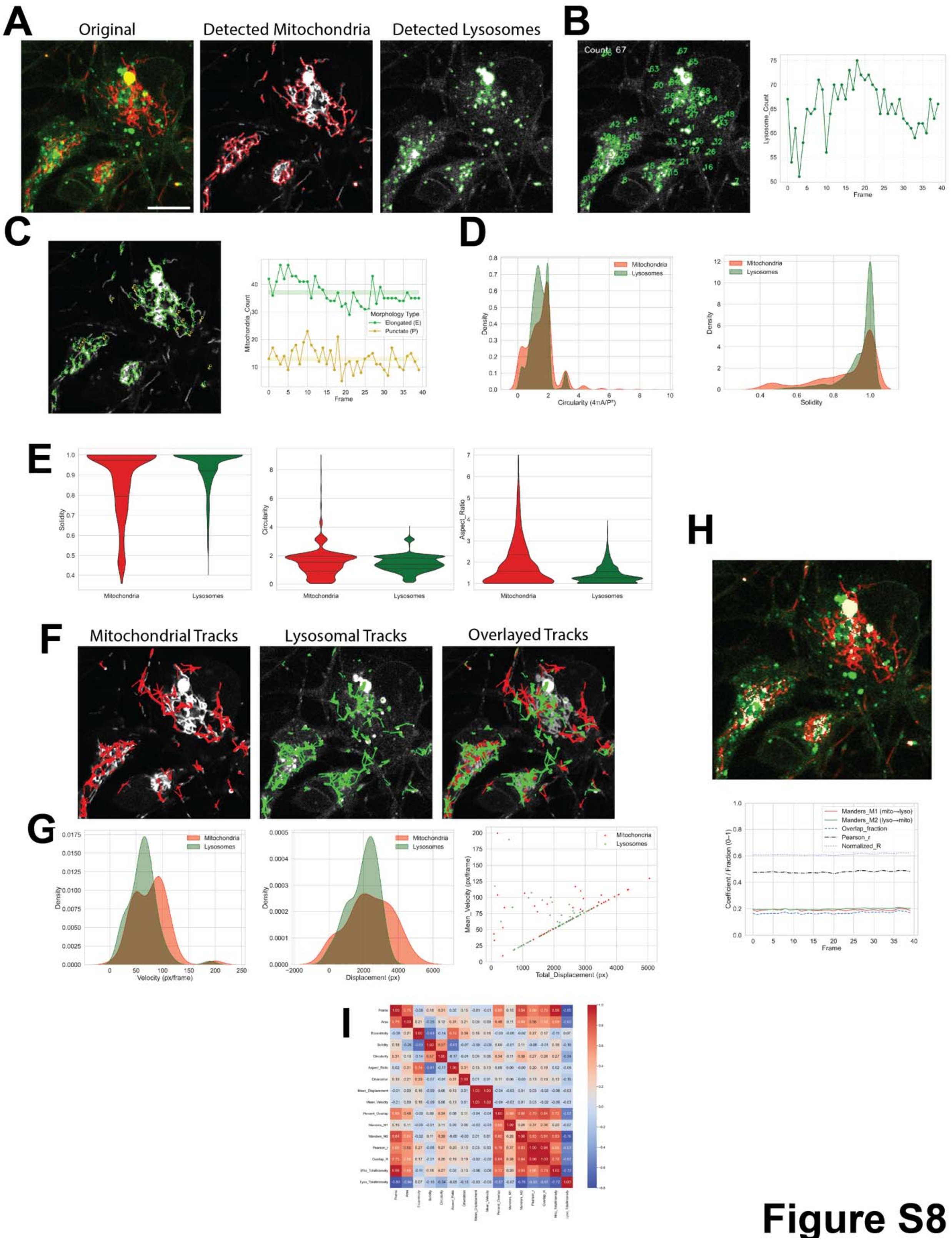

Figure S8

bioRxiv preprint doi: https://doi.org/10.1101/2025.07.19.665650; this version posted February 2, 2026. The copyright holder for this preprint (which was not certified by peer review) is the author/funder, who has granted bioRxiv a license to display the preprint in perpetuity. It is made available under aCC-BY 4.0 International license.

Supplementary Figures 1-8. AutoMorphoTrack outputs of other analyzed image stacks.

Functional Steps in the AutoMorphoTrack workflow were run on additional image stacks to verify the validity and applicability of the package. (**A**) Shows channel isolation, thresholding, and organelle segmentation. (**B**) Organelle segmentation enables the quantification of lysosomes across multiple frames. (**C**) The segmented mitochondrial channel is used to quantify mitochondrial morphology (Elongated vs Punctate). (**D** and **E**) Analysis of organelle morphology and structural profiling across different measures. (**F** and **G**). The trajectory of organelles and the cumulative path taken is quantified and visualized, along with displacement and velocity. (**H**) The colocalization of mitochondria and lysosomes is visualized and quantified across various approaches. (**I**) A comparative analysis of the image stack is conducted across all the measures discussed earlier.
